# Structural Characteristics and Efficacy of Polysaccharides from Fenjiu Vinasse in Alleviating DSS-Induced Ulcerative Colitis

**DOI:** 10.3390/foods15142575

**Published:** 2026-07-22

**Authors:** Huiqin Guo, Yi Chen, Jiangying Shi, Shuhua Shan, Zhuoyu Li, Nifei Wang, Xiushan Dong

**Affiliations:** 1 Institute of Biotechnology, Biomedicine and Health Laboratory in Shanxi Province, The Key Laboratory of Chemical Biology and Molecular Engineering of Ministry of Education, Shanxi University, Taiyuan 030002, China; guohuiqin@sxbqeh.com.cn (H.G.);; 2 Shanxi Bethune Hospital, Shanxi Academy of Medical Sciences, Third Hospital of Shanxi Medical University, Tongji Shanxi Hospital, Taiyuan 030032, China; 3 Department of General Surgery, Shanxi Bethune Hospital, Shanxi Academy of Medical Sciences, Third Hospital of Shanxi Medical University, Tongji Shanxi Hospital, Taiyuan 030032, China

**Keywords:** Fenjiu vinasse, polysaccharides, structural characterization, intestinal flora, ulcerative colitis

## Abstract

Vinasse, a major by-product of Fenjiu liquor production, is enriched with polysaccharides. However, their structural characteristics and potential anti-inflammatory functions remain unclear. In this study, polysaccharides from Fenjiu vinasse (VPS) were extracted and fractionated into seven sub-fractions (VPS-20 to VPS-80) via sequential alcohol precipitation. The molecular weight (Mw) of the fractions gradually decreased with increasing ethanol concentration. Monosaccharide composition analysis revealed that VPS and its fractions were mainly composed of glucose, mannose, and galactose. Among them, VPS-30 exhibited the most potent anti-inflammatory activity in LPS-induced RAW 264.7 macrophages by significantly reducing NO, IL-1β, IL-6, and TNF-α production. Structural characterization by methylation analysis and NMR spectroscopy indicated that VPS-30 contained six major glycosidic linkages, including 2,3-Ara*f*, T-Man*p*, 4-Man*p*, 3-Gal*p*, 4-Glc*p*, and 4,6-Man*p*, and its detailed structure was predicted. Furthermore, in DSS-induced ulcerative colitis (UC) mice, VPS-30 markedly alleviated colonic inflammation, restored intestinal barrier integrity by upregulating MUC-2, occludin, and ZO-1, and reshaped gut microbiota diversity, particularly by increasing beneficial genera such as *Limosilactobacillus*, *Butyricicoccus*, and *Ligilactobacillus*. Overall, these findings suggest that Fenjiu vinasse polysaccharides exert significant anti-inflammatory effects and may serve as promising functional food candidates for UC intervention.

## 1. Introduction

Ulcerative colitis (UC) is a chronic, relapsing inflammatory bowel disease characterized by abdominal pain, diarrhea, and rectal bleeding. The pathogenesis of UC is complex and multifactorial, including genetic factors [1], immune response disorders [2], intestinal barrier damage [3], and intestinal flora ecological disorders [4]. Current clinical management relies heavily on pharmacological interventions, including corticosteroids, 5-aminosalicylic acid, biologic therapies, probiotics and immunosuppressants. However, these therapies are often associated with adverse effects, limited long-term efficacy in some patients, and high relapse rates. Optimizing treatment strategies for UC has become an issue of critical urgency that demands immediate attention.

Increasing attention has been directed toward natural bioresources with therapeutic potential. As the solid residue of fermented grains, vinasse is a major by-product of the brewing process [5], with an annual production that exceeds 100 million tons [6]. Owing to its high moisture content, untreated vinasse is prone to rapid spoilage, leading to environmental concerns as well as a waste of valuable bioresources. Importantly, compositional analyses have indicated that vinasse contains various components, including polysaccharides, flavonoids, amino acids, proteins, and organic acids, which exhibit antioxidative and anti-inflammatory activities [7]. Despite these bioactive properties, the utilization of vinasse remains limited. Current applications are mainly restricted to animal feed, biogas production, composting, edible fungus cultivation and secondary fermentation processes [8], with animal feeding being the predominant use. Therefore, the high-value utilization of vinasse is still insufficiently explored, and its conversion into functional bioactive products may provide both environmental and economic benefits.

Among the bioactive components of vinasse, polysaccharides have attracted considerable attention. Polysaccharides are widely distributed natural macromolecules in plants and animals and have attracted significant scientific interest due to their structural diversity, low toxicity, and multiple biological activities, including anti-inflammatory [9], antioxidant [10], and intestinal homeostasis-rebuilding properties [11]. Importantly, vinasse-derived polysaccharides have shown promising biological activities in previous studies. For example, Liu et al. reported that ethanol- and water-soluble polysaccharides from Baijiu vinasse activated the NRF2/HO-1 axis to protect against alcoholic liver injury [12]. Similarly, Cui et al. isolated GPS-2B from Baijiu vinasse, which alleviated colitis through regulating gut microbiota and suppressing inflammatory signaling pathways [13]. However, the detailed structural characteristics and therapeutic potential of polysaccharides extracted from Fenjiu vinasse in ameliorating UC have not yet been fully elucidated.

To address this gap, ethanol precipitation is widely used for polysaccharide fractionation due to its simplicity and mild conditions. This method separates polysaccharides based on differential solubility in ethanol, in which higher molecular weight fractions precipitate at lower ethanol concentrations, enabling efficient fractionation. Previous studies have shown that ethanol fractionation can alter polysaccharide composition and bioactivity [14]. For instance, polysaccharide fractions from *Zizania latifolia* and *Asparagus* exhibited distinct biological activities after sequential ethanol precipitation [15,16]. In addition, Zhang et al. reported that *Schisandra Sphenanthera* polysaccharide fractions precipitated with 80% ethanol significantly increased short-chain fatty acid production [17].

Based on this, Fenjiu vinasse polysaccharides (VPS) and seven fractions (VPS-20 to VPS-80) were obtained via sequential ethanol precipitation. VPS-30 was identified as the most active fraction in lipopolysaccharide (LPS)-induced RAW 264.7 macrophages. Its structure was then characterized using chromatographic and spectroscopic methods, and its therapeutic effect was validated in a dextran sulfate sodium (DSS)-induced ulcerative colitis model. These results support the potential of Fenjiu vinasse polysaccharides as functional ingredients for UC intervention.

## 2. Materials and Methods

### 2.1. Materials

Vinasse was supplied by Shanxi Xinghuacun Fen Wine Factory (Shanxi, Fenyang, China), and the murine monocyte–macrophage RAW 264.7 cells were obtained from China Center for Type Culture Collection (GDC0143, Hubei, Wuhan, China). DMEM and fetal bovine serum were purchased from GIBCO (Grand Island, NY, USA, Cat. No. 11965092). The nitric oxide (NO) kit (Cat. S0021S) was from Biyuntian Biotechnology (Shanghai, China). ELISA kits for interleukin-1 beta (IL-1β, Cat. MM-0040M1), interleukin-6 (IL-6, Cat. MM-1011M2) and tumor necrosis factor-alpha (TNF-α, Cat. MM-0132M1) were acquired from Enzyme Immunity Industry (Suqian, China). Trizol reagent (Cat. SM129-02), All-in-One First-Strand cDNA Synthesis Kit II (with dsDNase) (Cat. SM134) and 2× SYBR Green qPCR MasterMixII (Cat. SM143) kits were obtained from Seven Biotech (Beijing, China). Glucose (CAS: 50-99-7), galacturonic acid (CAS: 91510-62-2) and polysaccharide molecular weight (Mw) standards were provided by Solaibao Biotech (Beijing, China). All other chemicals and solvents were obtained from Tianjin StarMark Science and Technology Development (Tianjin, China).

### 2.2. Polysaccharide Extraction and Fractionation

Vinasse and distilled water were mixed at a ratio of 1:10 and extracted at 90 °C. The supernatant was collected by centrifugation. Four volumes of 95% ethanol were added to the supernatant, and the precipitate was collected by centrifugation. Proteins were removed using the Sevage method (chloroform:n-butanol = 4:1, *v*/*v*, repeated extraction to remove protein impurities), and the sample was then freeze-dried to obtain VPS. Anhydrous ethanol was added to the VPS aqueous solution to a final concentration of 20% (*v*/*v*). The supernatant was collected by centrifugation, and then anhydrous ethanol was added to the supernatant to a final concentration of 30% (*v*/*v*). The precipitate was collected by centrifugation and freeze-dried to obtain VPS-30. Following the same method, anhydrous ethanol was added to the supernatant to final concentrations of 40%, 50%, 60%, 70%, and 80%, respectively, yielding the VPS-40, VPS-50, VPS-60, VPS-70 and VPS-80 fractions.

### 2.3. Structural Characteristics

#### 2.3.1. Chemical Composition Analysis

The protein concentration was quantified using the Bicinchoninic Acid (BCA) protein assay kit (P0012, Beyotime, Shanghai, China), according to the manufacturer’s instructions.

The total neutral sugar content was determined using the phenol–sulfuric acid method. Briefly, glucose was used as the standard to construct a calibration curve. Samples were reacted with phenol and concentrated sulfuric acid, followed by incubation in a boiling water bath, and the absorbance was measured at 490 nm using a UV–visible spectrophotometer. The neutral sugar content was calculated based on the standard curve.

The uronic acid content was measured using the m-hydroxydiphenyl colorimetric method. Briefly, galacturonic acid was used as the standard to construct a calibration curve. Samples were reacted with borate–sulfuric acid reagent under heating conditions, followed by the addition of m-hydroxydiphenyl reagent, and the absorbance was measured at 520 nm using a microplate reader. The uronic acid content was calculated according to the standard curve.

#### 2.3.2. Mw Determination

The Mw distribution of the polysaccharides was determined using an efficient differential liquid chromatography system equipped with a Waters Ultrahydrogel column (7.8 × 300 mm, 6 µm, Water Corporation, Milford, MA, USA) and a high-sensitivity refractive index detection unit (RID-20A). Polysaccharide samples were prepared at a concentration of 5 mg/mL in 0.1 M sodium nitrate solution, filtered through a 0.22 µm membrane, and 10 µL of the filtrate was injected for analysis. The column temperature was maintained at 40 °C, and 0.1 M sodium nitrate solution (Sigma-Aldrich, St. Louis, MO, USA) was used as the mobile phase at a flow rate of 0.5 mL/min.

#### 2.3.3. Monosaccharide Composition

The monosaccharide composition was analyzed by ion chromatography using a Thermo Fisher Scientific ICS 5000+ system (Thermo Fisher Scientific, Waltham, MA, USA) equipped with an electrochemical detector. Separation was performed on a Dionex CarboPac PA20 column (150 × 3.0 mm, 10 µm, Thermo Fischer Scientific, Sunnyvale, CA, USA) at 30 °C. Before analysis, polysaccharide samples were hydrolyzed with 2 M trifluoroacetic acid at 121 °C for 2 h, followed by nitrogen drying. The residues were repeatedly washed with methanol, re-dried, and finally re-dissolved in ultrapure water for analysis. A 5 µL aliquot was injected for each run. The mobile phases consisted of (A) ultrapure water, (B) 0.1 M NaOH, and (C) 0.1 M NaOH containing 0.2 M sodium acetate. The flow rate was maintained at 0.5 mL/min. The elution gradient was programmed as follows: 0–26 min, A/B/C (95:5:0, *v*/*v*); 26–42 min, A/B/C (85:5:10, *v*/*v*); 42–52 min, A/B/C (85:5:10, *v*/*v*); 52.1–60 min, A/B/C (60:0:40, *v*/*v*); followed by re-equilibration to initial conditions.

#### 2.3.4. Fourier Transform Infrared (FT-IR) Spectroscopy

Lyophilized VPS and VPS-30 samples were finely ground with potassium bromide, pressed into transparent pellets, and analyzed using an FT-IR spectrometer (Thermo Fisher Scientific, Waltham, MA, USA). Spectral data were recorded over a wavenumber range of 4000 to 400 cm^−1^.

#### 2.3.5. Methylation Analysis

(1) Methylation Reaction: First, 3 mg of polysaccharide was dissolved completely in 0.5 mL of dimethyl sulfoxide. Then, 20 mg of sodium hydroxide (NaOH) was added and stirred for 2 h. Next, 0.3 mL of iodomethane (CH_3_I) was slowly added dropwise with continued stirring for 2.5 h. The solution was transferred to a 25 mL flat-bottomed test tube, and the reaction vessel was rinsed three times with 2 mL of CH_3_I, combining the washes. Then, 5 mL of ultrapure water was added, the mixture was shaken vigorously, and the aqueous phase was discarded. This extraction process was repeated three times. Using a rubber-bulb pipette, the organic phase was collected and passed through an anhydrous sodium sulfate (Na_2_SO_4_) column. The column was further washed twice with 0.5 mL of dichloromethane (CH_2_Cl_2_), and the eluate was collected in a 5 mL ampoule. (2) Hydrolysis and Reduction: First, 0.5 mL of 4 M trifluoroacetic acid (TFA) was added to the ampoule bottle, which was then sealed and hydrolyzed at 100 °C for 6 h. After cooling, the solvent was evaporated under a nitrogen (N_2_) stream. Then, 0.3 mL of ultrapure water was added, followed by 1 drop of 5% aqueous ammonia and 4 mg of sodium borodeuteride (NaBD_4_). The mixture was vortexed and stirred at room temperature for 12 h. (3) The resulting polysaccharide solution was subjected to acetylation. (4) Gas Chromatography–Mass Spectrometry (GC-MS) Analysis: The sample was analyzed using a Shimadzu GC-MS system equipped with an RXI-5 SIL MS capillary column (30 m × 0.25 mm × 0.25 μm). The temperature was initially set at 120 °C and ramped at 3 °C/min to a final temperature of 250 °C. Both the injection port and detector were maintained at 250 °C. Helium served as the carrier gas, delivered at a constant flow rate of 1.0 mL/min under isobaric conditions.

#### 2.3.6. Nuclear Magnetic Resonance (NMR) Spectroscopy

VPS-30 was fully dissolved in deuterium oxide (D_2_O) (40 mg/mL). An aliquot of 0.5 mL was dispensed into a standard NMR tube for spectral acquisition. One-dimensional proton nuclear magnetic resonance (^1^H NMR) and carbon-13 nuclear magnetic resonance (^13^C NMR), as well as two-dimensional proton–proton correlation spectroscopy (^1^H-^1^H COSY), nuclear Overhauser effect spectroscopy (NOESY), proton–carbon heteronuclear single quantum coherence (^1^H-^13^C HSQC), and heteronuclear multiple bond correlation (HMBC) spectra were obtained at 25 °C using a high-field nuclear magnetic resonance spectrometer.

### 2.4. Assessment of Cell Viability

RAW 264.7 macrophages were maintained in DMEM supplemented with 10% (*v*/*v*) fetal bovine serum, 100 U/mL penicillin, and 100 μg/mL streptomycin. Cells were incubated at 37 °C in a humidified atmosphere containing 5% CO_2_. For the viability assay, each well of a 96-well plate was inoculated with 1 × 10^4^ cells. After overnight incubation, the medium was replaced with fresh DMEM containing 2 μg/mL LPS, and the cells were incubated for an additional 24 h. Subsequently, cells were treated with various concentrations of polysaccharide samples (VPS, VPS-20, VPS-30, VPS-40, VPS-50, VPS-60, VPS-70, and VPS-80) for another 24 h. Cell viability was assessed using the MTT (C0009S, Beyotime, Shanghai, China) assay according to the standard protocols.

### 2.5. NO Content Assay

RAW 264.7 macrophages were plated in 12-well plates at 1 × 10^5^ cells/well and subjected to the treatments outlined in Section 2.4. Following incubation, the NO levels in the culture supernatants were determined using a commercial NO assay kit, in accordance with the manufacturer’s guidelines.

### 2.6. RNA Extraction, cDNA Synthesis and qPCR

Total RNA was isolated with Trizol reagent following the supplier’s instructions. One microgram of purified RNA was reverse transcribed into cDNA using the All-in-One First-Strand cDNA Synthesis Kit II (TransGen Biotech Co., LTD, Beijing, China). Quantitative PCR was carried out with a 2× SYBR Green qPCR MasterMix (Thermo Fisher Scientific, Waltham, MA, USA) on a real-time PCR instrument. PCR amplification was carried out with an initial denaturation step at 95 °C for 30 s, followed by 40 cycles consisting of 95 °C for 15 s (denaturation) and 60 °C for 30 s (annealing/extension). GAPDH was included as the endogenous control. Primer sequences are listed in Table 1.

### 2.7. Animal Experiments

The healthy six-week-old C57BL/6J male mice were obtained from the Beijing Vital River Laboratory Animal Technology Co., Ltd. (Beijing, China). and acclimated in the China Institute of Radiation Protection under standardized conditions (26 ± 1 °C temperature, 60 ± 5% humidity, 12 h light/dark cycle) with free access to food and water. All animal experiments were approved by the Ethics Committee of the Drug Safety Evaluation Center in the China Institute for Radiation Protection (Approval Code: CIRP-IACUC-(R)2024067; Approval Date: 31 October 2024) and were conducted in accordance with institutional guidelines and ethical standards for animal care and use. After a one-week acclimation period, animals were randomized into 4 groups (*n* = 10): control, model, VPS and VPS-30 groups (*n* = 10). DSS (2.5%) was administered in the drinking water to all groups except the control throughout the 7 days to induce acute colitis. After successful induction of colitis, treatments were administered daily by oral gavage for another 7 days. Control and DSS model groups received saline (10 mL/kg), whereas VPS and VPS-30 groups received the respective polysaccharide at 200 mg/kg body weight. Clinical parameters, including body weight, food and water consumption, stool consistency, and fecal occult blood, were recorded daily. Following overnight fasting, mice were euthanized. Colonic length was measured, and intestinal content was harvested for microbiome profiling. Colon tissues were immersed in 4% paraformaldehyde for pathological analysis, with remaining specimens stored at −80 °C for further analyses.

### 2.8. Enzyme-Linked Immunosorbent (ELISA) Assay

ELISA kits were used to determine the concentrations of IL-1β, IL-6, and TNF-α in serum samples and in the supernatants of RAW 264.7 murine macrophages after treatment with different concentrations of VPS and VPS-30, according to the manufacturer’s instructions.

### 2.9. Histological Analysis

Collected colonic specimens were immersed in 4% paraformaldehyde solution for 24 h, dehydrated through an ethanol series, and embedded in paraffin. Sections (4 μm) were cut, deparaffinized, and stained with hematoxylin and eosin (H&E). Morphological features were then evaluated and imaged using a Nikon Eclipse Ci upright microscope (Nikon Corporation, Tokyo, Japan). Image acquisition was performed using the Nikon Digital Sight DS-FI2 imaging system (Nikon Corporation, Tokyo, Japan) and analyzed with NIS-Elements F software (Ver. 4.30.00, Nikon Instruments Inc., Tokyo, Japan).

### 2.10. Immunofluorescence Staining

Immunofluorescence assays for MUC-2 (Proteintech, Wuhan, China, Cat No. 27675-1-AP), occludin (Proteintech, Wuhan, China, Cat No. 27260-1-AP) and ZO-1 (Proteintech, Wuhan, China, Cat No. 21773-1-AP) were performed by Servicebio Technology Co., Ltd. (Wuhan, China). Briefly, colon tissue sections were placed in antigen repair solution, blocked, incubated with the corresponding primary antibody, and observed microscopically after dichloroaniline color development. Quantitative analysis of fluorescence intensity was performed using Image-Pro Plus 6.0 software.

### 2.11. 16S rRNA Gene Sequencing Analysis

Microbial DNA was isolated from colonic contents using the E.Z.N.A.^®^ Stool DNA Kit (Omega Bio-Tek, Norcross, GA, USA, Cat. No. D4015-02) in accordance with the manufacturer’s instructions. The V3-V4 regions of the bacterial 16S rRNA gene were amplified by PCR on a GeneAmp^®^ 9700 thermocycler (Applied Biosystems, Foster City, CA, USA) using primer pair 338F (5′-ACTCCTACGGGAGGCAGCAG-3′) and 806R (5′-GGACTACHVGGGTWTCTAAT-3′). After purification, amplicons were normalized, pooled, and sequenced in a 2 × 300 bp paired-end run on an Illumina MiSeq platform at Metware Biotechnology Co., Ltd. (Wuhan, China). Sequencing reads were processed and analyzed through the Metwar Cloud Platform (Platform version: 2026 stable version; supporting bioinformatics tools: FastQC v0.11.9, Trimmomatic v0.39, QIIME2 v2024.10, DADA2 v1.26, R v4.3.1) (https://cloud.metware.cn, accessed on 20 May 2026).

### 2.12. Statistical Analysis

Data were analyzed using GraphPad Prism 8 and MestReNova 9.0. Results were expressed as mean ± standard deviation of three independent experiments. Differences were considered statistically significant at *p* < 0.05.

## 3. Results

### 3.1. Chemical Composition Analysis

Extraction yields and compositional profiles of VPS and its seven ethanol-fractionated sub-fractions are detailed in Table 2. Neutral sugars accounted for over 60% of each fraction, with VPS-60 exhibiting the highest content at 93.90 ± 4.19%. Protein and uronic acid levels remained below 9% and 4%, respectively.

**Figure 1:**
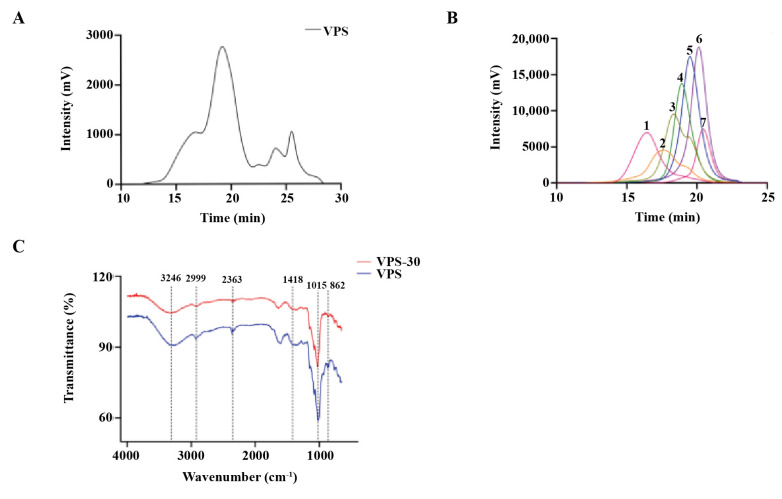
Mw and infrared absorption spectra of VPS components showing the reduction in Mw with increasing buffer polarity. (**A**) Mw of VPS. (**B**) Molecular weight of VPS components (From left to right 1–7: VPS-20, VPS-30, VPS-40, VPS-50, VPS-60, VPS-70, VPS-80). (**C**) Infrared absorption spectrum of VPS and VPS-30.

Polysaccharide bioactivities often tightly correlate with Mw [18]. According to Figure 1 and Table 2, the Mw decreased gradually with increasing ethanol concentrations in the order of VPS-20 (566.29 kDa), VPS-30 (111.97 kDa), VPS-40 (50.13 kDa), VPS-50 (21.16 kDa), VPS-60 (11.96 kDa), VPS-70 (6.51 kDa), and VPS-80 (4.28 kDa).

The monosaccharide components were detected and analyzed through ion chromatography. As shown in Table 3 and Figure S1, VPS was mainly composed of the following substances: mannose, glucose, galactose, xylose, arabinose, galacturonic acid and rhamnose. Their molar percentages were 6.26, 75.85, 3.61, 3.48, 6.38, 0.84 and 1.01, respectively. Ethanol fractionation altered both monosaccharide composition and abundance, though glucose, mannose, and galactose remained predominant across all sub-fractions. It is worth noting that the monosaccharide composition of VPS-30 is similar to that of the parent VPS, with the molar percentages of mannose, glucose, galactose, xylose and arabinose being 6.67, 76.09, 4.23, 3.97 and 6.99, respectively. These findings were comparable to Cui et al.’s characterization of DGPS-2B, a 37.3 kDa water-soluble polysaccharide from Chinese liquor vinasse, whose composition (xylose:arabinose:glucose:galactose:rhamnose = 55.5:36.6:3.7:3.0:1.2) underscored the diversity of vinasse-derived polysaccharides [13]. These results suggest that ethanol fractionation mainly alters molecular size rather than fundamental monosaccharide composition, implying that bioactivity differences are more likely due to structural organization rather than sugar types.

### 3.2. Effects of VPS on Macrophage Viability

**Figure 2:**
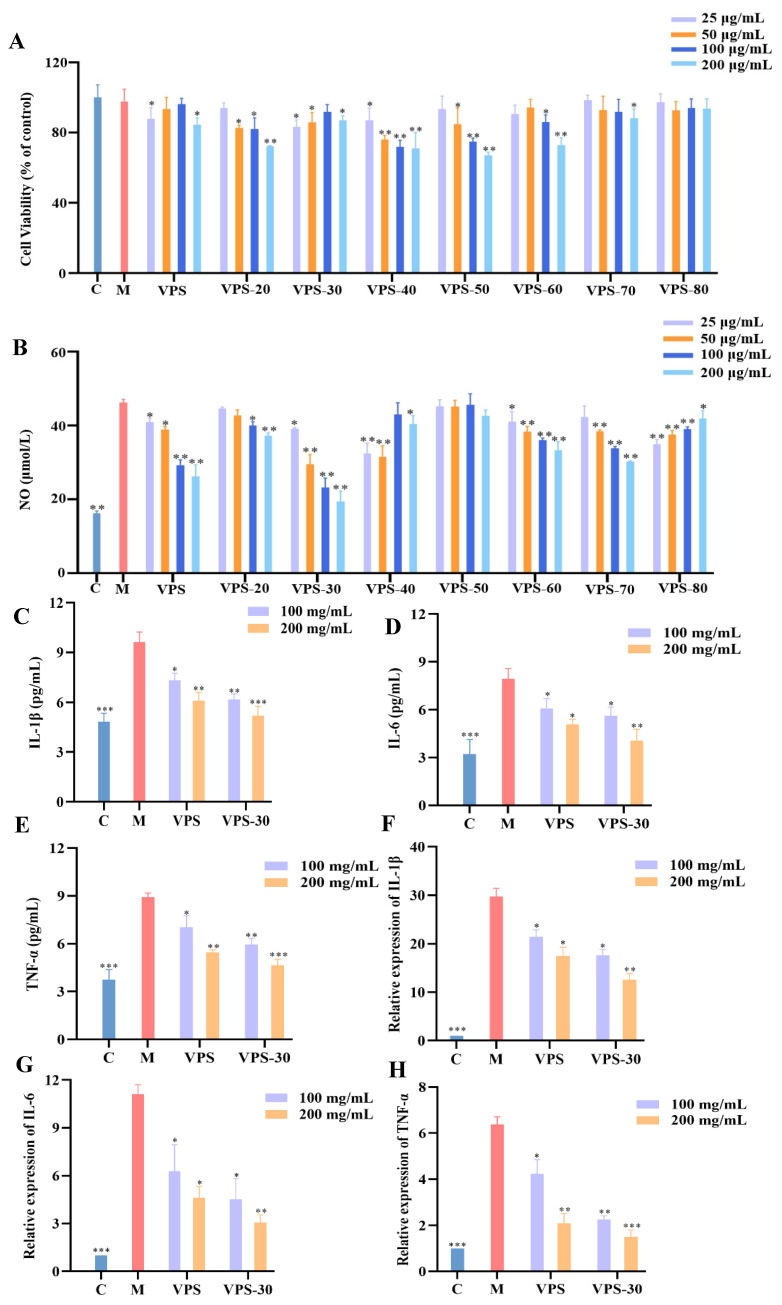
Low cytotoxicity and significant anti-inflammatory effects of VPS-30 components in LPS induced RAW 264.7 macrophages. (**A**) Effect of VPS components on cell viability of LPS-induced RAW 264.7 macrophages. (**B**) Effect of VPS components on NO secretion by LPS-induced RAW 264.7 macrophages. (**C**–**E**) Inhibitory effect of VPS and VPS-30 on the production of IL-1β (**C**), IL-6 (**D**), and TNF-α (**E**) in LPS-induced RAW264.7 macrophages. (**F**–**H**) Suppressive effect of VPS and VPS-30 on mRNA levels of IL-1β (**F**), IL-6 (**G**) and TNF-α (**H**) in LPS-induced RAW264.7 macrophages. (**A**) * *p* < 0.05, ** *p* < 0.01 vs. control group; (**B**–**H**) * *p* < 0.05, ** *p* < 0.01, *** *p* < 0.001 vs. model group.

MTT assays were conducted to evaluate the effects of VPS and its fractions on the proliferation of LPS-stimulated RAW 264.7 macrophages. Treatment with VPS, VPS-30, VPS-70, or VPS-80 at concentrations of 25, 50, 100, and 200 µg/mL for 24 h maintained survival rates exceeding 80% across all groups (Figure 2A), indicating low cytotoxicity under the tested conditions.

### 3.3. Inhibition of NO and Pro-Inflammatory Cytokines Release by VPS

Studies have shown that LPS activation of RAW 264.7 cells triggers the release of the pro-inflammatory mediator NO, which amplifies oxidative and inflammatory stress [19,20]. As depicted in Figure 2B, LPS markedly elevated the production of NO in macrophages. VPS, VPS-30, VPS-60, VPS-70 and VPS-80 all inhibited NO release compared with the model group. Particularly, VPS-30 exhibited the most potent, dose-dependent inhibition, followed by VPS, leading to the selection of these two fractions for subsequent analyses.

ELISA was further used to evaluate the release of pro-inflammatory mediators in the culture supernatant. The results demonstrated that VPS and VPS-30 substantially reduced secreted IL-1β, IL-6, and TNF-α in the culture supernatant (Figure 2C–E), with VPS-30 showing superior efficacy. Concordantly, qPCR analysis revealed parallel decreases in IL-1β, IL-6, and TNF-α mRNA expression (Figure 2F–H). Together, these findings confirm that VPS-30 exhibits superior anti-inflammatory efficacy in LPS-induced RAW 264.7 macrophages. Collectively, these findings demonstrate that VPS-30 exerts the strongest anti-inflammatory effect by suppressing key inflammatory mediators in activated macrophages.

### 3.4. FT-IR Spectral Characterization of VPS-30

FT-IR spectroscopy was utilized to characterize the functional groups present in VPS and VPS-30. As illustrated in Figure 1C, both polysaccharide samples (VPS and VPS-30) displayed highly similar absorption profiles, indicating that ethanol fractionation did not introduce new functional groups or significantly alter the primary chemical structure. A broad and intense peak around 3246 cm^−1^ corresponded to the O-H stretching vibration, reflecting extensive hydrogen bonding typical of polysaccharides. The peak observed at approximately 2999 cm^−1^ was attributed to the C-H stretching vibration of methyl groups [21]. The absorption observed at 2363 cm^−1^ likely arose from intramolecular C-H vibrations. A distinct absorption peak at 1652 cm^−1^ was indicative of the C=O stretching vibration [22], suggesting the presence of minor uronic or carbonyl-containing groups. The peak near 1418 cm^−1^ represented the C-H bending vibration [23]. The region between 1250 and 950 cm^−1^ suggested the presence of pyranose ring conformations in both VPS and VPS-30 [24]. Additionally, the band observed at 862 cm^−1^ confirmed the presence of α-glycosidic linkages and the skeletal vibrations of pyranose structures [21]. This indicates that ethanol fractionation does not significantly alter the fundamental chemical backbone, suggesting that differences in activity are not due to new functional groups but to structural rearrangements.

### 3.5. Methylation Profiling of VPS-30

To investigate the glycosidic linkage patterns and quantify the molar distribution of sugar residues, methylation analysis was performed on both VPS and VPS-30 [25]. The analytical data for VPS and VPS-30 are summarized in Table 4 and Figure S2. In VPS, six types of partially methylated alditol acetates were identified, including 2,3-linked arabinofuranose (2,3-Ara*f*), terminal mannopyranose (T-Man*p*), 4-linked mannopyranose (4-Man*p*), 3-linked galactopyranose (3-Gal*p*), 4-linked glucopyranose (4-Glc*p*), and 4,6-linked mannopyranose (4,6-Man*p*), with respective molar ratios of 5.62:3.97:3.73:1.00:79.08:6.60. VPS-30 exhibited the same residue types, but with altered molar proportions: 1.50:5.78:4.47:3.80:80.06:4.40. While the monosaccharide constituents remained unchanged, VPS-30 showed a higher proportion of T-Man*p*, 4-Man*p*, and 3-Gal*p* compared to VPS, along with a reduction in 2,3-Ara*f*, 4-Glc*p*, and 4,6-Man*p*. These variations suggest that structural modifications occurred during fractionation, potentially influencing the bioactivity of the polysaccharide. These changes suggest that fractionation modifies the glycosidic linkage distribution, which may contribute to the enhanced bioactivity of VPS-30.

### 3.6. NMR Analysis of VPS-30

**Figure 3:**
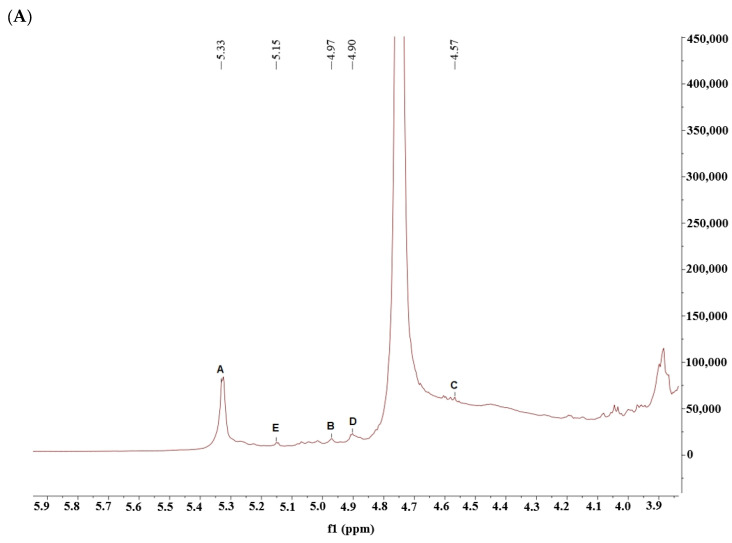

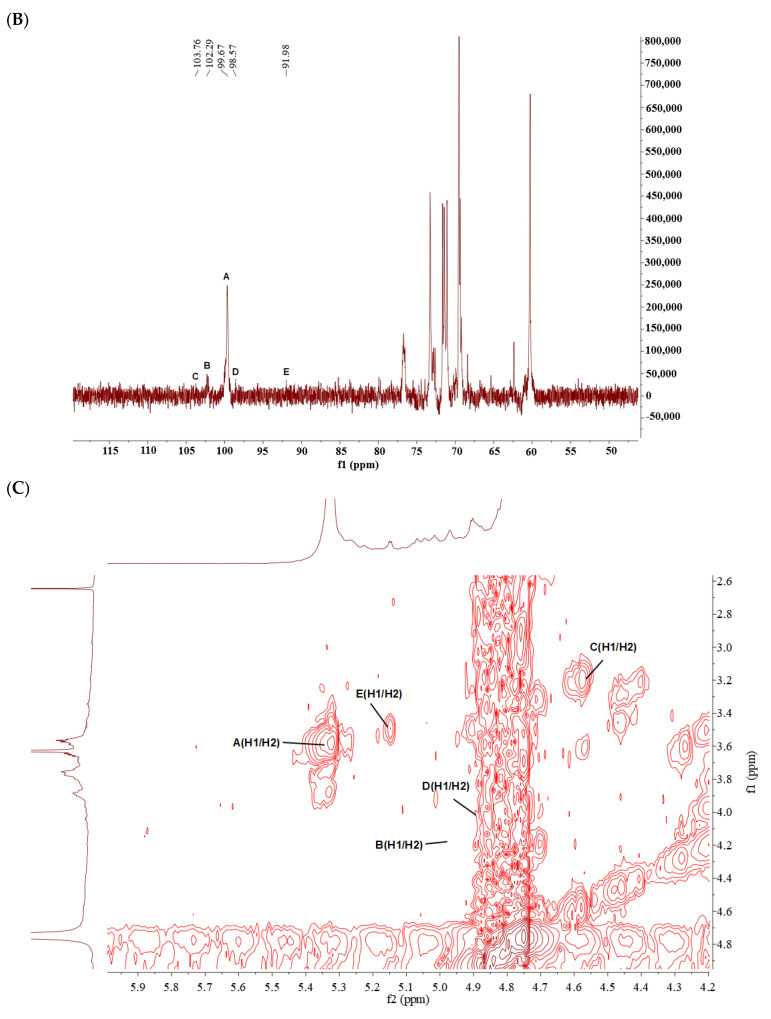

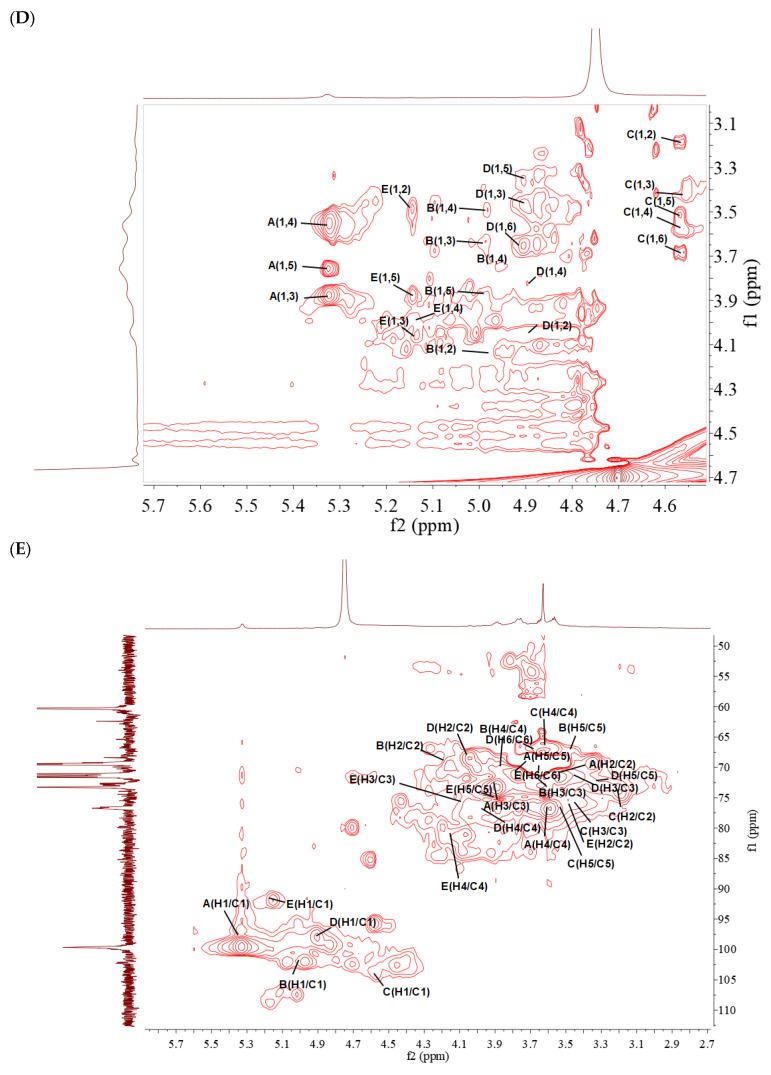

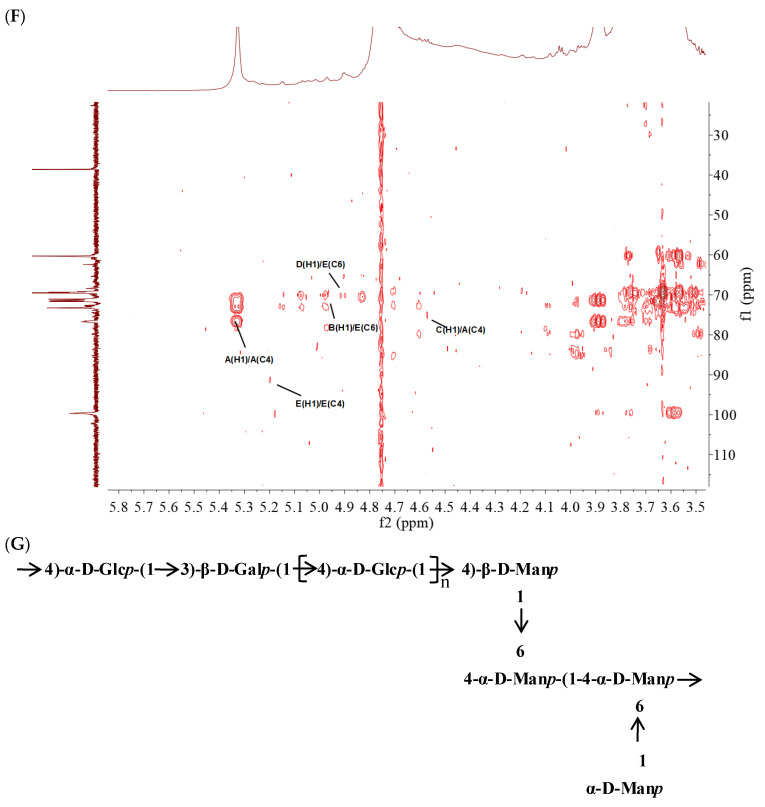
NMR spectra of VPS-30. (**A**) ^1^H-NMR spectrum of VPS-30. (**B**) ^13^C -NMR spectrum of VPS-30. (**C**) ^1^H-^1^H COSY spectrum of VPS-30. (**D**) TCOSY spectrum of VPS-30. (**E**) HSQC spectrum of VPS-30. (**F**) HMBC spectrum of VPS-30. (**G**) The predicted structural representation of VPS-30.

To comprehensively characterize the structural features of VPS-30, both one-dimensional (1D) and two-dimensional (2D) NMR spectroscopy were performed. The ^1^H and ^13^C NMR spectra of VPS-30 are presented in Figure 3A and Figure 3B, respectively. In the ^1^H-NMR spectrum, anomeric proton signals of sugar residues typically resonate in the δ 4.5–5.5 ppm range. Five distinct signals were observed in this region for VPS-30 at δ 5.33 ppm (peak **A**), δ 4.97 ppm (peak **B**), δ 4.57 ppm (peak **C**), δ 4.90 ppm (peak **D**) and δ 5.15 ppm (peak **E**), suggesting the presence of five types of anomeric protons (Figure 3A and Figure S3A). In the ^13^C-NMR spectrum, the heterotopic carbon signal of sugar residues typically appears between δ90 and 110 ppm [23]. For VPS-30, five prominent anomeric carbon signals were detected at δ 99.67, δ 102.29 ppm, δ 103.76 ppm, δ 98.57 ppm, and δ 91.98 ppm (Figure 3B and Figure S3A), respectively. Notably, no signals were observed between δ 82–88 ppm in the ^13^C-NMR spectra of VPS-30, a region typically associated with C3 and C5 of furanose rings, indicating that the sugar units in VPS-30 predominantly exist in pyranose form. Further structural elucidation was achieved through heteronuclear single quantum coherence (HSQC) spectra, which provided correlations between the proton and carbon chemical shifts of residues **A**, **B**, **C**, **D** and **E**.

Proton chemical shifts were further elucidated using proton–proton correlation spectroscopy (^1^H-^1^H COSY) and nuclear Overhauser effect spectroscopy (NOESY) spectra. The detailed chemical shift assignments are summarized in Table 5. For residue **A**, the anomeric proton signal was observed at δ 5.33 ppm, with the corresponding anomeric carbon at δ 99.67 ppm (Figure 3A,B). The sequential proton signals were H-2 at δ 3.57 ppm, H-3 at δ 3.88 ppm, H-4 at δ 3.55 ppm, and H-5 at δ 3.75 ppm, as determined by COSY and total correlation spectroscopy (TOCSY) spectra (Figure 3C,D and Figure S3C,D). In addition, the C-1 to C-6 chemical shift (99.67 ppm) of **A** was obtained from HSQC (Figure 3E). Therefore, based on the methylation results and relevant literature, residue **A** was identified as →4)-α-D-Glc*p*-(1 → [26,27,28]. For residue **B**, the identified proton signals were H-1 (4.97 ppm), H-2 (4.14 ppm), H-3 (3.63 ppm), H-4 (3.49 ppm), and H-5 (3.85 ppm), with its anomeric carbon appearing at 102.29 ppm. Therefore, based on the methylation results and relevant literature, residue **B** was identified as α-D-Man*p*-(1→ [28,29]. For residue **C**, the identified proton signals were H-1 (4.57 ppm), H-2 (3.19 ppm), H-3 (3.42 ppm), H-4 (3.58 ppm), H-5 (3.51 ppm) and H-6 (3.68 ppm), with its anomeric carbon appearing at 103.76 ppm. Therefore, based on the methylation results and relevant literature, residue **C** was identified as →3)-β-D-Gal*p*-(1→ [30]. For residue **D**, the identified proton signals were H-1 (4.90 ppm), H-2 (4.05 ppm), H-3 (3.47 ppm), H-4 (3.94 ppm), H-5 (3.36 ppm) and H-6 (3.65 ppm), with its anomeric carbon appearing at 98.57 ppm. Therefore, based on the methylation results and relevant literature, residue **D** was identified as →4)-β-D-Man*p*-(1→ [31]. For residue **E**, the identified proton signals were H-1 (5.15 ppm), H-2 (3.48 ppm), H-3 (4.07 ppm), H-4 (3.99 ppm), H-5 (3.88 ppm) and H-6 (3.62 ppm), with its anomeric carbon appearing at 91.98 ppm. Therefore, based on the methylation results and relevant literature, residue **E** was identified as →4,6)-α-D-Man*p*-(1→ [32].

The heteronuclear multiple bond correlation (HMBC) and NOESY spectra (Figure S3E) revealed inter-residue and intra-residue correlations. In the HMBC spectrum (Figure 3F), there were overlapping peaks between H-1 of residue **A** (at 5.33 ppm) and C-4 (at 76.75 ppm). There was a correlation between H-1 of residue **B** (at 4.97 ppm) and C-6 of residue **E** (at 69.58 ppm). Furthermore, the H-1 site of residue **C** (4.57 ppm) exhibited correlation with the C-4 site of residue **A** (76.75 ppm). Similarly, the H-1 site of residue **D** (4.90 ppm) showed correlation with the C-6 site of residue **E** (69.58 ppm), and the H-1 site of residue **E** (5.15 ppm) demonstrated correlation with its own C-4 site (78.28 ppm). The NOESY spectrum (Figure S3E) further confirmed the residue correlations. The structural formula of VPS-30 is shown in Figure 3G.

### 3.7. VPS-30 Attenuates DSS-Induced Ulcerative Colitis in Mice

**Figure 4:**
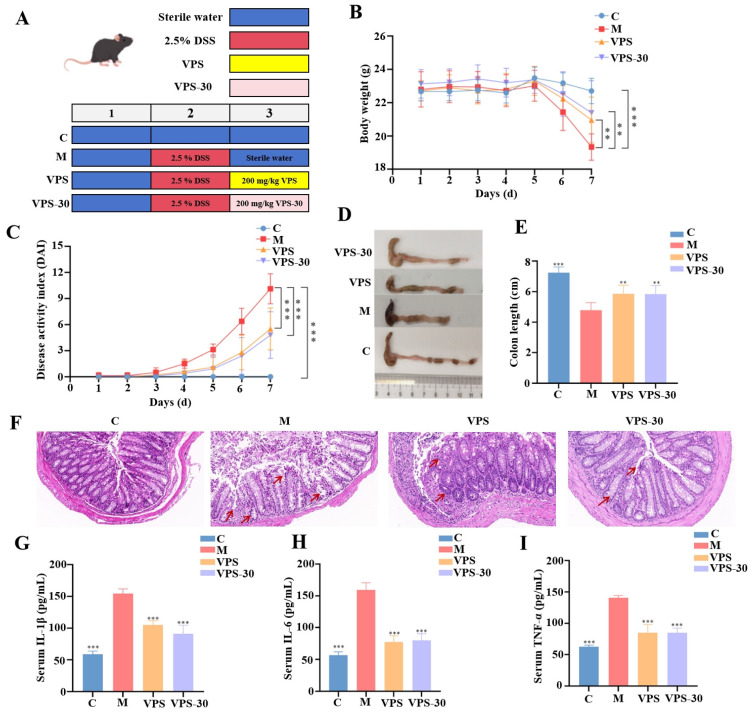
VPS-30 alleviates DSS-induced ulcerative colitis by improving disease activity and reducing inflammatory responses. VPS-30 alleviates DSS-induced ulcerative colitis by improving disease activity and reducing inflammatory responses. (**A**) Schematic diagram of the experimental procedure. (**B**) Effects of VPS-30 on body weight in mice with UC. (**C**) Effects of VPS-30 on DAI in mice with UC. (**D**,**E**) Effects of VPS-30 on colon length in mice with UC. (**F**) Photomicrographs of H&E-stained sections (200 μm) of colons in mice. (**G**–**I**) Level of pro-inflammatory cytokines in serum, including IL-1β (**G**), IL-6 (**H**), and TNF-α (**I**). ** *p* < 0.01, *** *p* < 0.001 vs. model group.

To evaluate the therapeutic effects of VPS and VPS-30 on the progression of ulcerative colitis, C57BL/6J mice were administered 2.5% DSS in their drinking water for 14 days to induce the ulcerative colitis model, with daily oral gavage of saline or polysaccharide (200 mg/kg). The experimental design is illustrated in Figure 4A. DSS administration provoked significant body weight loss compared to the healthy control, whereas mice treated with VPS or VPS-30 exhibited a more gradual decline (Figure 4B). Consistent with these observations, the disease activity index (DAI), combining weight loss, stool consistency, and fecal blood, was markedly elevated in the DSS group but partially normalized by both polysaccharide interventions (Figure 4C). Inflammation, colonic hyperemia, and edema leading to a shortened colon were typical features of ulcerative colitis. Therefore, we further measured colon length in the mice. Our results showed that colon shortening, a hallmark of DSS-induced injury, was pronounced in untreated mice but significantly alleviated in the VPS and VPS-30 groups, with VPS-30 showing superior preservation of colon length (Figure 4D,E).

Subsequently, histopathological evaluation via HE staining (Figure 4F) was carried out to assess the morphological alterations in the colonic sections of mice. We found intact mucosal architecture in the control, whereas DSS induced epithelial erosion, crypt loss, goblet cell depletion, and inflammatory infiltrates. Both VPS and VPS-30 restored epithelial integrity, reduced inflammatory infiltrates, and preserved goblet cell populations, with the most pronounced effect observed in the VPS-30 cohort. In addition, elevation of pro-inflammatory cytokines is a key feature of DSS colitis [33]. Regulating the secretion of pro-inflammatory factors is an important strategy to suppress the inflammatory response. Subsequently, circulating concentrations of pro-inflammatory cytokines were quantified in serum. As expected, systemic levels of IL-1β, IL-6, and TNF-α were markedly elevated in the model group. They were significantly reduced following VPS and VPS-30 administration (Figure 4G–I). These observations align with prior reports demonstrating that an acidic polysaccharide derived from Phellinus linteus mitigates DSS-induced ulcerative colitis in mice [6]. Collectively, these results indicate that VPS-30 effectively mitigates clinical and histopathological features of ulcerative colitis, preserving body weight, reducing disease activity, preventing colon shortening, and dampening systemic inflammatory responses. Together, these findings indicate that VPS-30 attenuates intestinal inflammation at both tissue and systemic levels.

### 3.8. VPS-30 Restores Mucosal Integrity and Tight Junction Protein Expression in DSS-Induced Colitis

**Figure 5:**
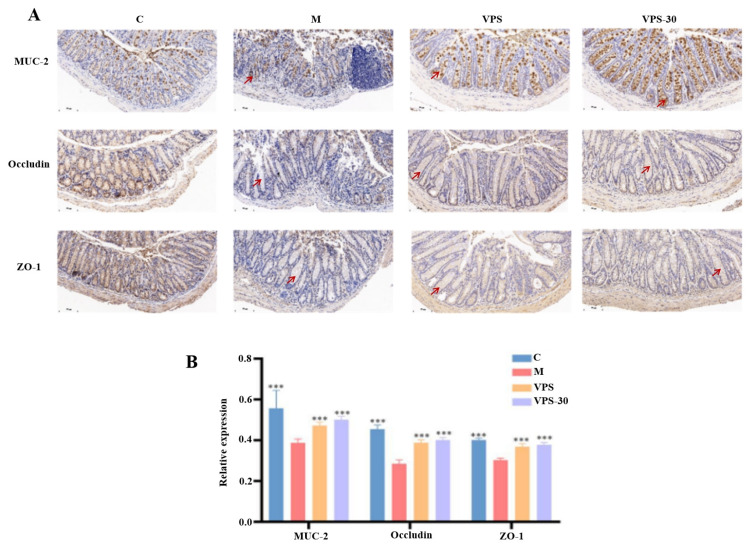
VPS-30 restores intestinal barrier function by enhancing MUC-2, occludin, and ZO-1 expression in DSS-induced colitis. (**A**) Immunofluorescence staining of MUC-2, occludin, and ZO-1 proteins on colonic sections (200 μm) was performed to assess barrier restoration. (**B**) Expression of MUC-2, occludin, and ZO-1 proteins was quantified using the Image J software 1.4.3.67. *** *p* < 0.001 vs. model group.

To assess barrier restoration, we performed immunofluorescence staining for MUC-2, occludin and ZO-1 proteins on colonic sections. As shown in Figure 5, DSS treatment markedly reduced MUC-2 staining and downregulated occluding and ZO-1 expression. Both VPS and VPS-30 interventions significantly upregulated these proteins, with VPS-30 exerting the most pronounced effect. This suggests that VPS-30 improves intestinal barrier integrity, which is a key mechanism in reducing colitis severity.

### 3.9. VPS-30 Enhanced Gut Microbial Diversity in DSS-Induced Colitis

**Figure 6:**
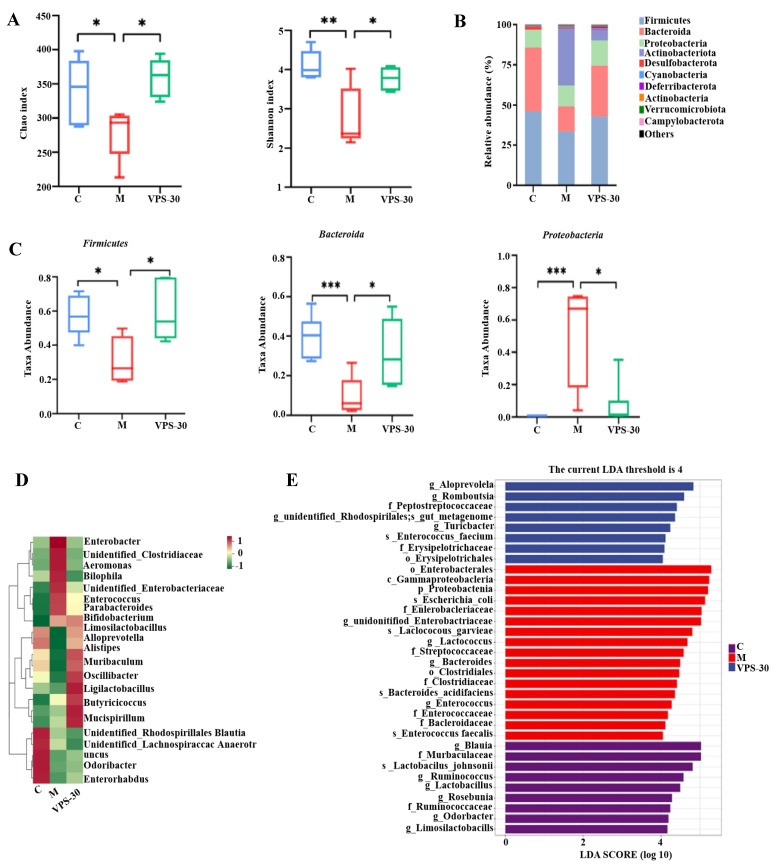
VPS-30 modulates gut microbiota composition and restores microbial balance in DSS-induced colitis. (**A**) Chao index and Shannon index. (**B**) Stacked bar chart of species relative abundance at the phylum level. (**C**) Significant intergroup differences were found in 3 phyla. (**D**) Heatmap of species abundance at the genus level. (**E**) LEfSe analysis. * indicates *p* < 0.05 compared with the model group, and ** indicates *p* < 0.01 compared with the model group. * *p* < 0.05, ** *p* < 0.01, *** *p* < 0.001 vs. model group.

To evaluate the impacts of VPS-30 on the gut microbiome, 16S rRNA sequencing was performed on fecal samples. Gut microbial richness and diversity were quantified using the Chao1 and Shannon indices. As shown in Figure 6A, DSS-induced colitis caused a significant decrease in both metrics, whereas VPS-30 treatment substantially elevated Chao1 and Shannon values. This demonstrates that VPS-30 effectively restores microbial community richness and diversity.

In addition, we detected changes in bacterial abundance at the phylum and genus levels by taxonomic unit analysis (Figure 6B,C). At the phylum level, VPS-30 triggered an overgrowth of *Bacteroidota* and *Clostridia* and a depletion of *Gammaproteobacteria*. At the genus level, VPS-30 markedly enriched *Muribaculum*, *Limosilactobacillus*, *Butyricicoccus*, and *Ligilactobacillus*. In brief, taxonomic profiling at the phylum and genus levels further demonstrated that VPS-30 reshaped the microbial landscape. Subsequently, LEfSe analysis identified discriminative taxa for each group. As illustrated in Figure 6D, the LDA threshold was set to 4 to identify significantly different species present in each group. At the genus level, the control group was enriched in *Alloprevotella*, *Romboutsia*, and *Turicibacter*; the DSS model exhibited overrepresentation of *Lactococcus*, *Bacteroides*, and *Enterococcus*; and the VPS-30 group was characterized by elevated *Blautia*, *Ruminococcus*, and *Lactobacillus*. These results indicate that VPS-30 functions as a prebiotic, selectively fostering beneficial microbial populations and reestablishing gut microbial homeostasis in ulcerative colitis.

## 4. Discussion

Vinasse, a major by-product of Baijiu production, is rich in polysaccharides with reported antioxidant and anti-inflammatory activities. However, their biological functions are highly dependent on structural heterogeneity. Ethanol precipitation is a widely used separation strategy based on solubility differences in ethanol–water systems. Polysaccharides with higher Mw and lower polarity preferentially precipitate at lower ethanol concentrations, while smaller or more polar fractions precipitate at higher ethanol levels. In this study, seven sub-fractions polysaccharides (VPS-20 to VPS-80) with different Mw were obtained through sequential alcohol precipitation. The Mw decreased with increasing ethanol concentrations. These trends aligned with previously reported graded-alcohol precipitations of *Panax Notoginseng* and *Panax ginseng C. A. Meyer* polysaccharides [24,34]. These findings confirm that ethanol fractionation is an effective strategy to generate structurally diverse polysaccharide populations for bioactivity screening.

The structure of polysaccharides is the basis of their biological activity. Previous studies have shown that specific glycosidic linkages and branching patterns significantly influence immunomodulatory activity. For example, Chen et al. isolated APS-A1 and APS-B1 from *Astragalus*, noting enhanced activity in APS-B1, possibly due to the presence of →6)-α-D-Gal*p*-(1→ and →5)-α-L-Araf-(1→ linkages in its backbone [35]. Similarly, Zhang et al. (2024) identified WPZP-2-1 and WPZP-2-2, two RG-I-type pectic polysaccharides containing →4)-α-D-Gal*p*A-(1→2)-α-L-Rha*p*-(1→ as their main chains and varying side chains of galactans and arabinogalactans, both of which demonstrated anti-inflammatory activity [36]. Based on these comparisons and the observed structural features, we hypothesize that the notable in vitro anti-inflammatory efficacy of VPS-30 may be attributed to its backbone configuration, specifically consisting of →4)-α-D-Glc*p*-(1→ with α-D-Man*p*-(1→ branches. This suggests that branching patterns and glycosidic linkages play a key role in determining the anti-inflammatory activity of polysaccharides.

We next attempted to evaluate the relieving effects of VPS-30 on ulcerative colitis. Impaired intestinal barrier function is considered a crucial factor in the exacerbation of UC. MUC-2, the principal gel-forming mucin, constitutes the backbone of the protective mucus layer [37], whereas tight junction proteins such as occludin and ZO-1 are critical to maintain epithelial barrier integrity [38]. Our results indicated that VPS-30 effectively mitigates DSS-induced mucus layer erosion and tight junction disruption, thereby reinforcing the epithelial barrier. These findings are consistent with previous reports demonstrating that a *Scutellaria baicalensis* polysaccharide ameliorated colitis by enhancing ZO-1, occludin, and claudin-5 expression and preserving barrier function [39]. Collectively, these results indicate that epithelial barrier protection is a key mechanism underlying the therapeutic effects of VPS-30 in UC.

Disruption of the gut microbial ecosystem is both a driver and consequence of UC [40]. Certain polysaccharides have been shown to ameliorate colitis by modulating microbial communities. For example, Enterococcus-derived polysaccharide promoted the proliferation of probiotic *Parabacteroides distasonis* F1-28, attenuating DSS-induced colitis [41]. Lycium polysaccharides suppressed conditional pathogens such as *Mucispirillum* and *Sutterella*, restoring gut health [42]. *Ficus carica* polysaccharides enrich beneficial taxa (e.g., Bacteroidetes and S24-7) while reducing *Escherichia* and *Clostridium*, thereby improving colonic inflammation [43]. In our study, we found that VPS-30 restored microbial richness and diversity, including *Bacteroidota*, *Muribaculum*, *Limosilactobacillus*, *Butyricicoccus* and *Ligilactobacillus*. *Bacteroidota* members are crucial for immune modulation and the maintenance of microbial equilibrium [44]. *Limosilactobacillus* attenuates pro-inflammatory cytokine secretion [45]. *Butyricicoccus*, a key butyrate producer, has been shown to inhibit NF-κB activation in colonic macrophages [46], and *Ligilactobacillus* suppresses opportunistic pathogens [45]. These findings suggest that microbiota modulation and SCFA-associated metabolic regulation are involved in the protective effects of VPS-30.

In brief, VPS and VPS-30 exhibited significant anti-inflammatory activities in vivo and in vitro. Their protective effects on DSS-induced UC appear to involve the suppression of pro-inflammatory cytokine release, preservation of epithelial barrier integrity, and modulation of gut microbial composition. Structurally, VPS-30 retained the key features of the parent VPS, namely α-glycosidic linkages and pyranose ring conformations. Methylation analysis identified six common residues (2,3-Ara*f*, T-Man*p*, 4-Man*p*, 3-Gal*p*, 4-Glc*p*, and 4,6-Man*p*), with 4-Glc*p* predominating in both samples. NMR data revealed a backbone of →4)-α-D-Glc*p*-(1→ units with α-D-Man*p*-(1→ branches, which likely constitute the functional core of VPS-30. These results underscore the promise of Fenjiu vinasse-derived polysaccharides as nutraceutical agents or adjunctive therapeutics for UC.

## 5. Innovation and Limitations

In this study, we first elucidated the detailed structure and therapeutic efficacy of polysaccharides extracted from Fenjiu vinasse in ameliorating UC. Despite the persuasive evidence, this study still has some limitations. Here, we elucidated the structure of VPS-30 (→4)-α-D-Glc*p*-(1→ backbone with Man*p* branches) and observed its bioactivities in vitro and in vivo. Based on the literature review, polysaccharides with similar structures possess anti-inflammatory activities. For example, Li et al. extracted and purified a homogeneous polysaccharide LBP with a molecular weight of 312 kDa from the bulb of the lily and conducted a chemical structure analysis on it. The results showed that LBP was a 1-4-linked mannan glycoprotein, containing repeating units of →4)-β-D-Man*p*-(1→4)-β-D-Man*p*-(1→4)-β-D-Man*p*-(1→4)-β-D-Glc*p*-(1→4)-β-D-Man*p*-(1→4)-β-D-Man*p*-(1→4)-β-D-Glc*p*-(1→4)-α-D-Glc*p*-(1→4)-β-D-Glc*p*-(1→4)-β-D-Glc*p*-(1→4)-β-D-Man*p*-(1→4)-β-D-Man*p*-(1→. Moreover, it exhibited good immunomodulatory effects in vitro [47]. In this study, the structure of the polysaccharide of VPS-30 (→4)-α-D-Glc*p*-(1→ backbone with Man*p* branches) presented a similar structure. It also exhibited anti-inflammatory activity.

The elucidation of the structure–activity relationship of VPS-30 polysaccharides remains in its infancy. Future work should investigate how this specific structure influences the immune response, promotes beneficial bacteria (e.g., as a prebiotic), and enhances mucus and tight junction protein production. Secondly, according to the existing literature and our results, most polysaccharides exert anti-inflammatory effects and gut barrier protection mainly through microbiota modulation. We should obtain the corresponding advantageous strains and conduct further verification through in vivo studies in the future.

In addition, although we observed changes in the gut microbiota, including an increase in SCFA-producing bacteria such as Butyricicoccus, the actual levels of SCFAs, particularly butyrate, were not measured. This represents an important limitation, as SCFAs are key metabolites linking gut microbiota modulation with intestinal inflammation and barrier function. Future studies should address SCFA profiling to better elucidate the microbiota–metabolite–host axis underlying the anti-inflammatory effects.

## Figures and Tables

**Table 1:** Primer sequence.

Gene	Primer Sequences
TNF-α	Forward: CGCTCTTCTGTCTACTGAACTTCGG Reverse: GTGGTTTGTGAGTGTGAGGGTCTG
IL-1β	Forward: CACTACAGGCTCCGAGATGAACAAC Reverse: TGTCGTTGCTTGGTTCTCCTTGTAC
IL-6	Forward: CTTCTTGGGACTGATGCTGGTGAC Reverse: AGTGGTATCCTCTGTGAAGTCTCCTC
GAPDH	Forward: ACCCACTCCTCCACCTTTGA Reverse: CTGTTGCTGTAGCCAAATTCGT

**Table 2:** Yield and chemical composition of VPS components.

Samples	Yield (%)	Neutral Sugar (%)	Uronic Acid (%)	Protein (%)	Mw (kDa)
VPS	1.5 ± 0.13	76.46 ± 2.01	2.96 ± 0.26	7.23 ± 0.25	-
VPS-20	14.21 ± 0.73	80.48 ± 7.25	2.72 ± 0.45	0.38 ± 0.25	566.29
VPS-30	12.25 ± 0.41	67.03 ± 3.01	2.36 ± 0.56	2.17 ± 1.08	111.97
VPS-40	10.45 ± 0.3	78.47 ± 5.69	3.82 ± 0.08	4.49 ± 1.21	50.13
VPS-50	13.26 ± 0.26	63.04 ± 6.47	2.00 ± 0.15	1.31 ± 0.26	21.16
VPS-60	10.49 ± 1.22	93.90 ± 4.19	3.77 ± 0.06	4.27 ± 0.37	11.96
VPS-70	7.2 ± 0.32	80.48 ± 8.54	2.49 ± 1.16	2.87 ± 0.41	6.51
VPS-80	6.3 ± 0.65	60.40 ± 3.52	3.11 ± 0.80	8.43 ± 0.71	4.28

**Table 3:** The monosaccharide composition of VPS components.

Samples	Monosaccharide Composition (mol%)						
	Man	Glc	Gal	Xyl	Ara	GalA	Rha
VPS	6.26	75.85	3.61	3.48	6.38	0.84	1.01
VPS-20	1.21	98.02	-	-	0.77	-	-
VPS-30	6.67	76.09	4.23	3.97	6.99	-	-
VPS-40	3.52	94.25	1.43	-	0.80	-	-
VPS-50	1.16	98.84	-	-	-	-	-
VPS-60	2.09	93.67	1.99	0.8	1.46	-	-
VPS-70	0.97	93.72	1.48	1.37	2.46	-	-
VPS-80	1.60	79.66	2.90	4.69	8.75	0.82	1.59

**Table 4:** The methylation analysis results of VPS and VPS-30.

Tim (min)		PMAA	Type of Linkage	Mass Fragments (*m*/*z*)	Molar Ratio (%)	
VPS	VPS-30				VPS	VPS-30
20.69	20.62	5-Me Ara	2,3-Ara*f*	55, 69, 87, 99, 117, 129, 140, 157, 173, 188	5.62	1.5
21.30	21.26	2,3,4,6-Me_4_Man	T-Man*p*	59, 71, 87, 113, 118, 129, 145, 157, 161, 162, 205	3.97	5.78
21.97	21.94	2,3,6-Me_3_Man	4-Man*p*	59, 71, 87, 102, 118, 129, 162, 173, 203, 233	3.73	4.47
23.30	23.29	2,4,6-Me_3_Gal	3-Gal*p*	59, 74, 87, 101, 118, 129, 143, 161, 174, 234, 277	1.00	3.80
23.63	23.62	2,3,6-Me_3_Glc	4-Glc*p*	59, 71, 87, 99, 118, 129, 142, 173, 203, 233	79.08	80.06
25.89	25.88	2,3-Me_2_Man	4,6-Man*p*	59, 74, 85, 102, 118, 127, 162, 171, 187, 201, 261	6.60	4.40

**Table 5:** ^1^H/^13^C chemical shift of VPS and VPS-30 sugar residues.

	Sugar Residues	H1/C1	H2/C2	H3/C3	H4/C4	H5/C5	H6/C6
**A**	→4)-α-D-Glc*p*-(1→	5.33/99.67	3.57/71.50	3.88/73.32	3.55/76.75	3.75/71.14	-
**B**	α-D-Man*p*-(1→	4.97/102.15	4.14/70.01	3.63/72.99	3.49/67.33	3.85/70.62	-
**C**	→3)-β-D-Gal*p*-(1→	4.57/103.69	3.19/74.09	3.42/76.31	3.58/66.87	3.51/74.45	3.68/64.15
**D**	→4)-β-D-Man*p*-(1→	4.90/98.28	4.05/68.51	3.47/71.39	3.94/76.49	3.36/71.52	3.65/64.16
**E**	→4,6)-α-D-Man*p*-(1→	5.15/91.86	3.48/76.49	4.07/77.71	3.99/78.28	3.88/73.18	3.62/69.58

A–E correspond to the sugar residues listed in the table. The bold letters were used only to facilitate the identification and labeling of the corresponding signals in the NMR spectra; therefore, no additional explanation is required.

## Data Availability

The original contributions presented in this study are included in the article/Supplementary Material. Further inquiries can be directed to the corresponding authors.

## Supplementary Materials

The following supporting information can be downloaded at: https://www.mdpi.com/article/10.3390/foods15142575/s1, Figure S1: The monosaccharide composition of VPS components; Figure S2: Total Ion chromatogram of PMAA of VPS and VPS-30, and the mass spectra of each PMAA. (A) Total Ion chromatogram of PMAA of VPS. (B) Total Ion chromatogram of PMAA of VPS-30. (C) The mass spectra of each PMAA; Figure S3: NMR spectra of VPS-30. (A) 1H-NMR spectrum. (B) ^13^C-NMR spectrum. (C) ^1^H-^1^H COSY spectrum. (D) TCOSY spectrum. (E) NOSEY spectrum.
