# Supplementary material for: Structural Characteristics and Efficacy of Polysaccharides from Fenjiu Vinasse in Alleviating DSS-Induced Ulcerative Colitis
Source: Foods. 2026 Jul 22;15(14):2575. doi: 10.3390/foods15142575 (PMC13408780; doi:10.3390/foods15142575)
Supplement: Supplementary file 1 [file foods-15-02575-s001.zip › foods-4370658-supplementary.pdf]

Supplementary data

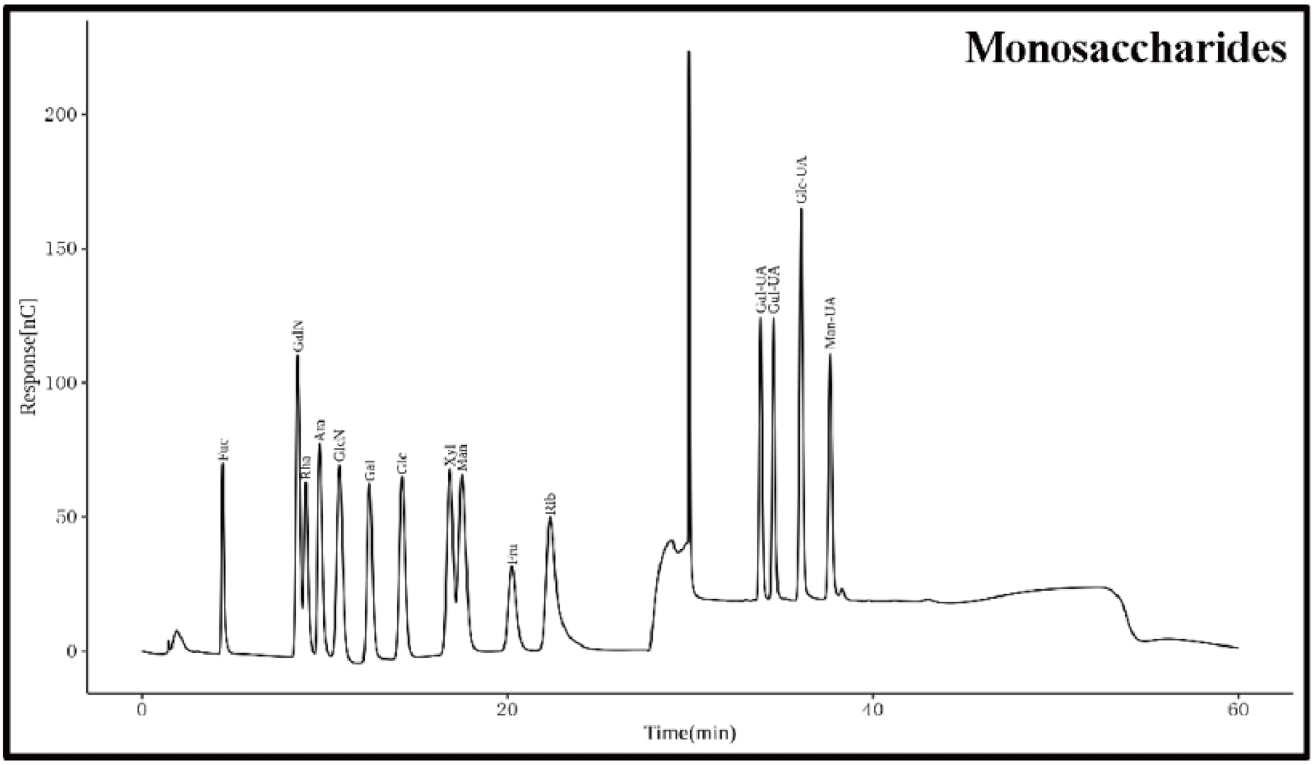

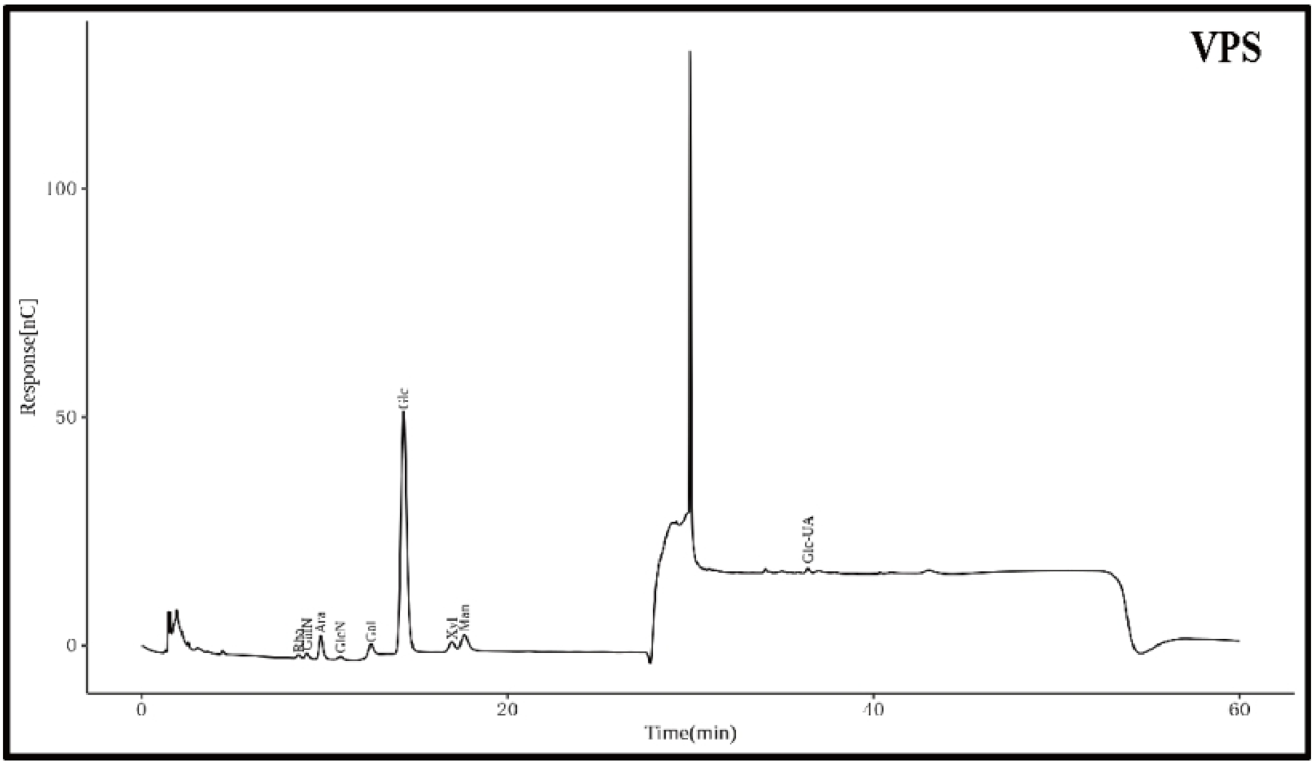

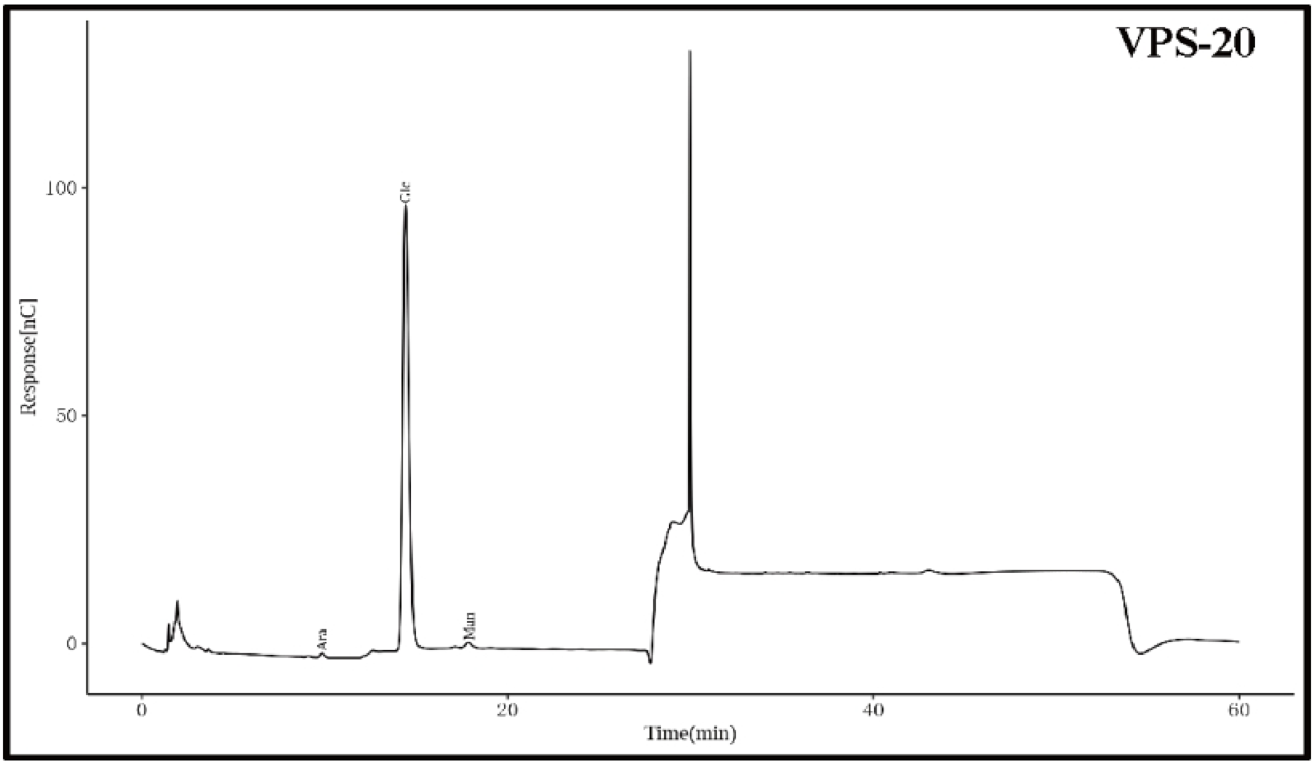
VPS-20
Response[nC]
Time(min)
Ara
Glc
Man

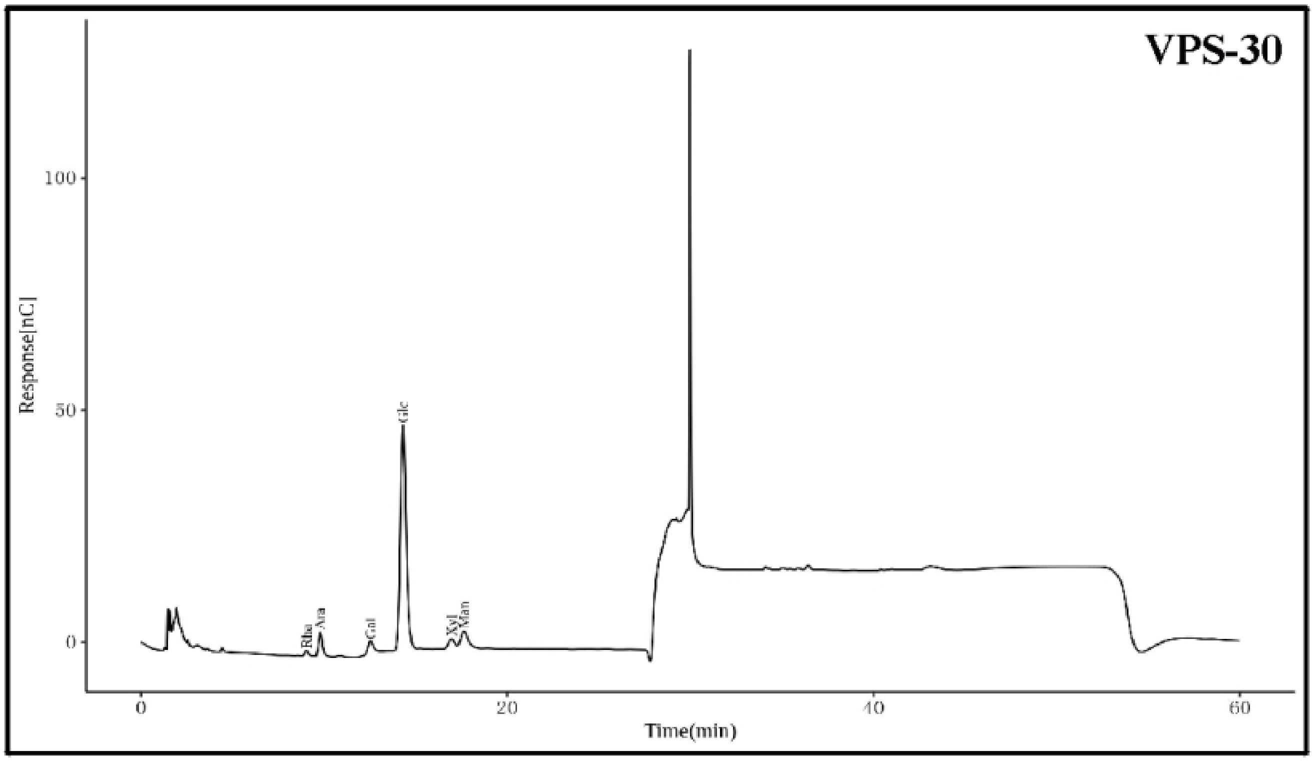
VPS-30
Response[nC]
Time(min)
Rha
Ara
Gal
Glc
Xyl
Man

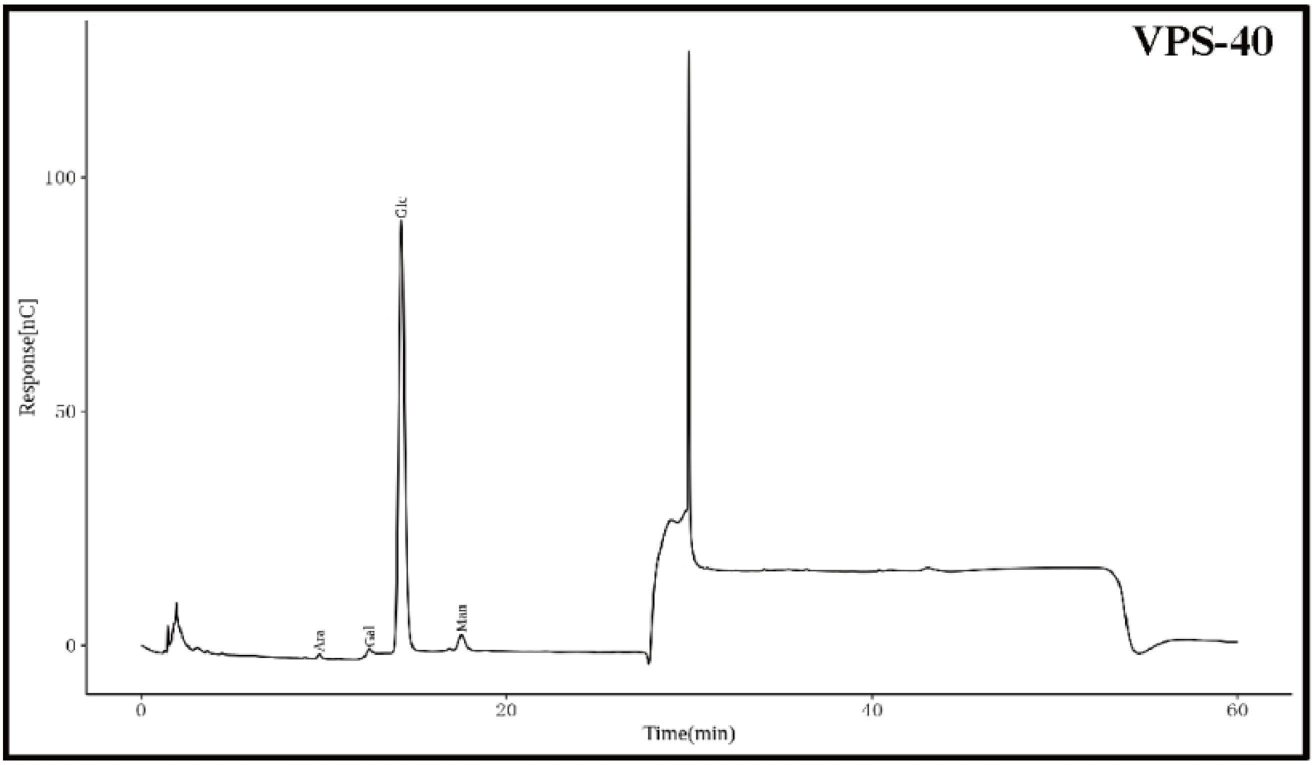
VPS-40
Response[nC]
Time(min)
Ara
Gal
Glc
Man

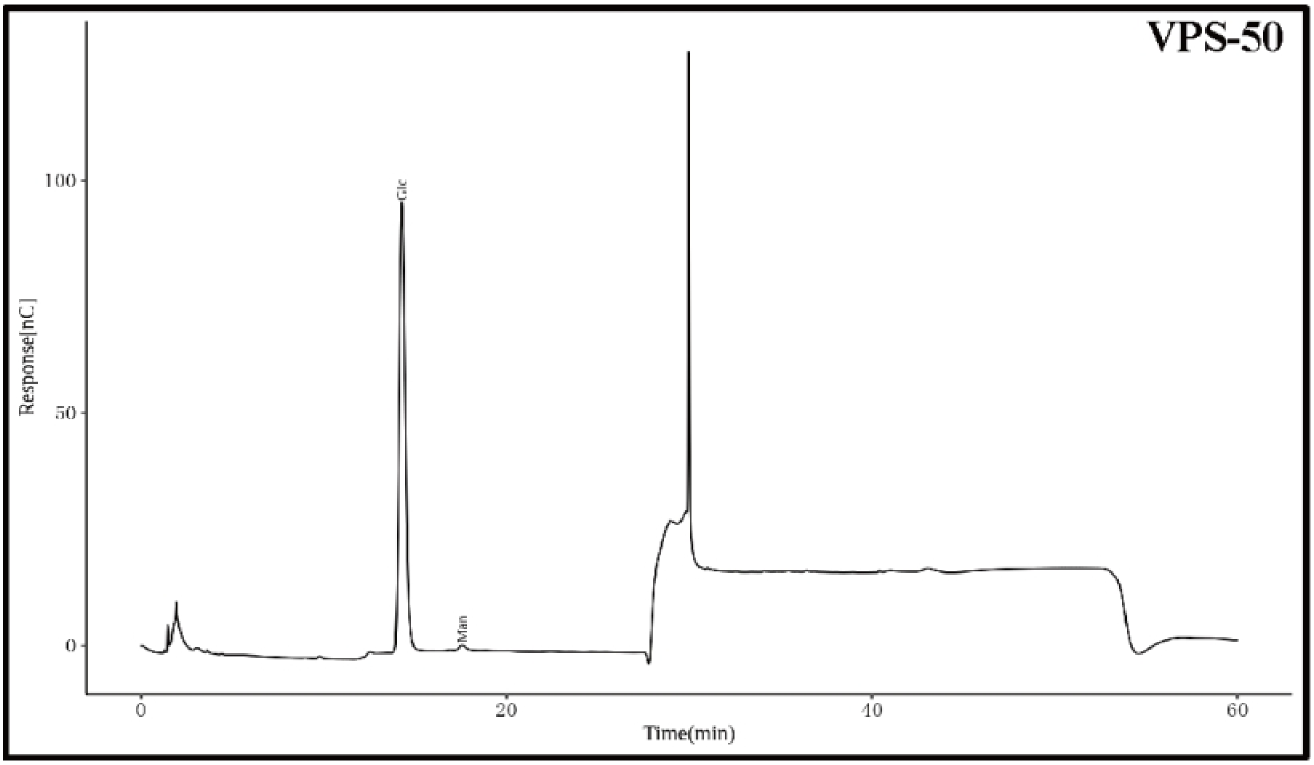
VPS-50
Response[nC]
Time(min)
Glc
Man

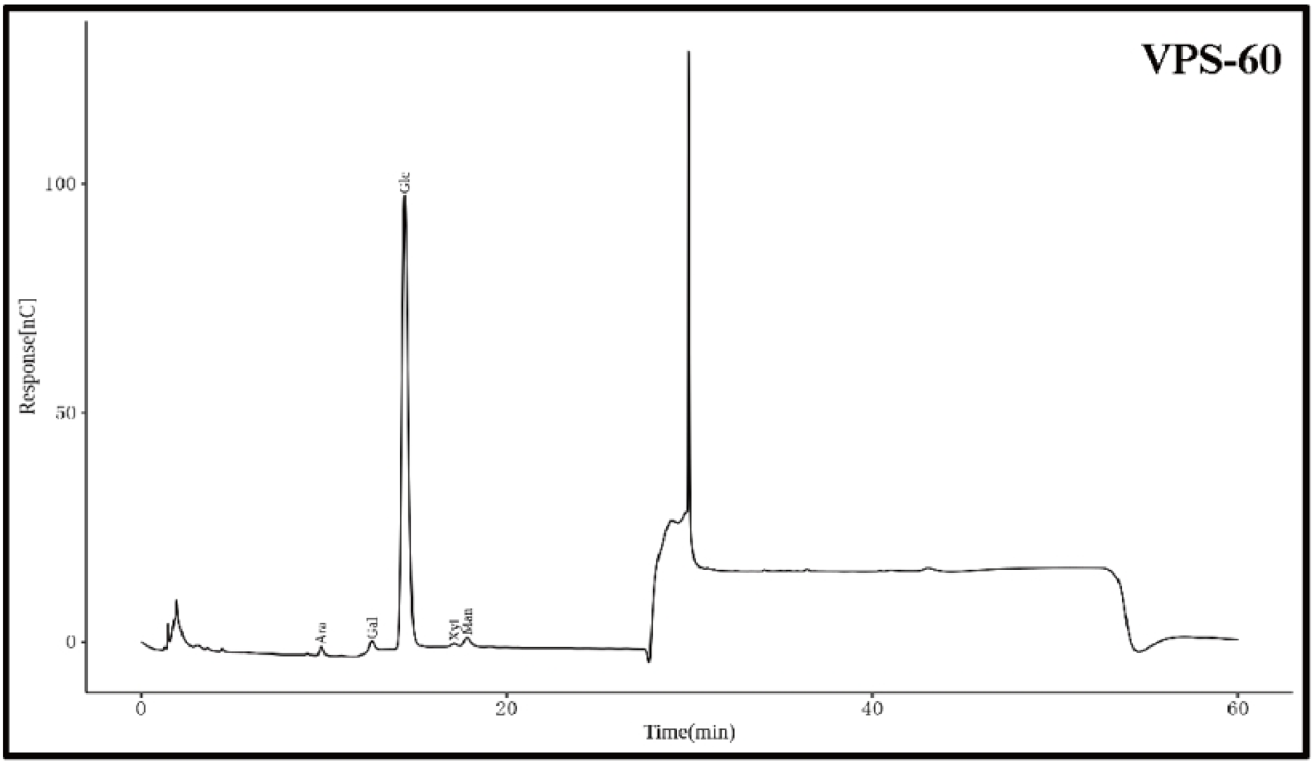
VPS-60
Response[nC]
Time(min)
Ara
Gal
Glc
Xyl
Man

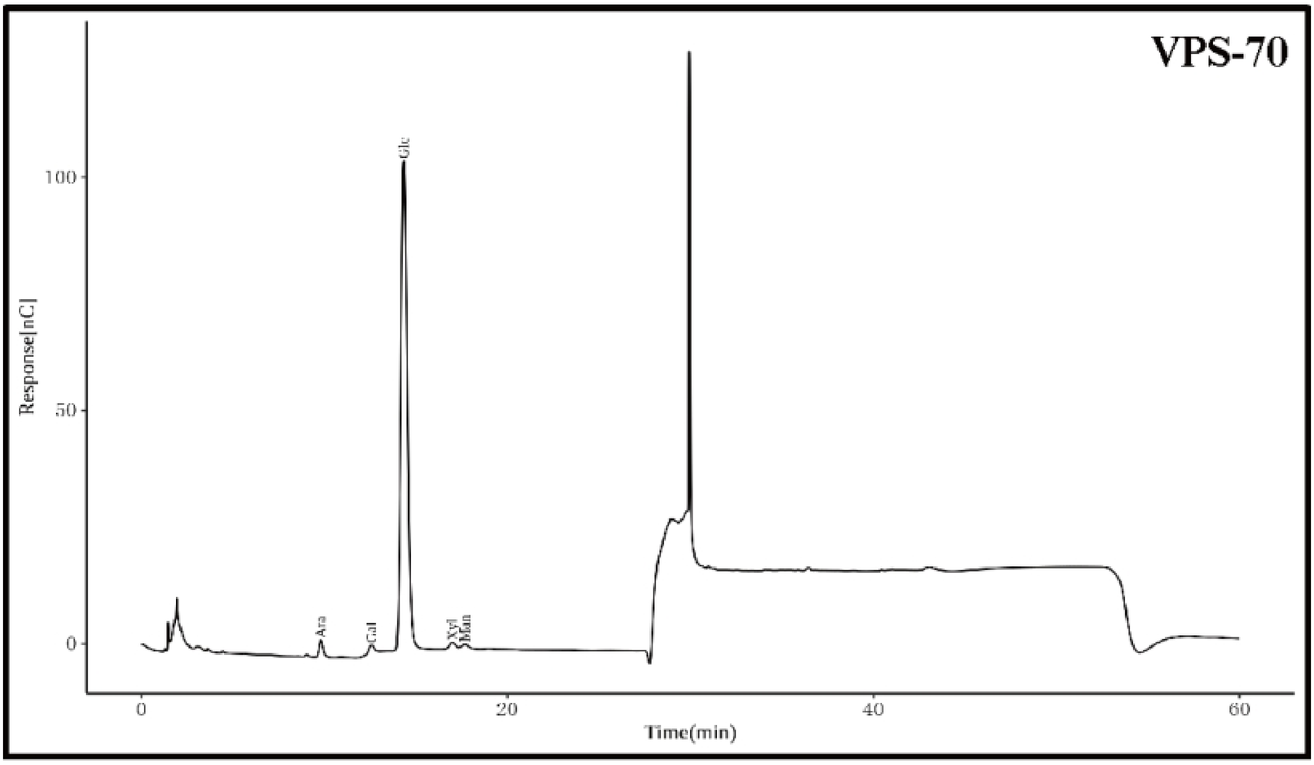
VPS-70
Response[nC]
Time(min)
Ara
Gal
Glc
Xyl
Man

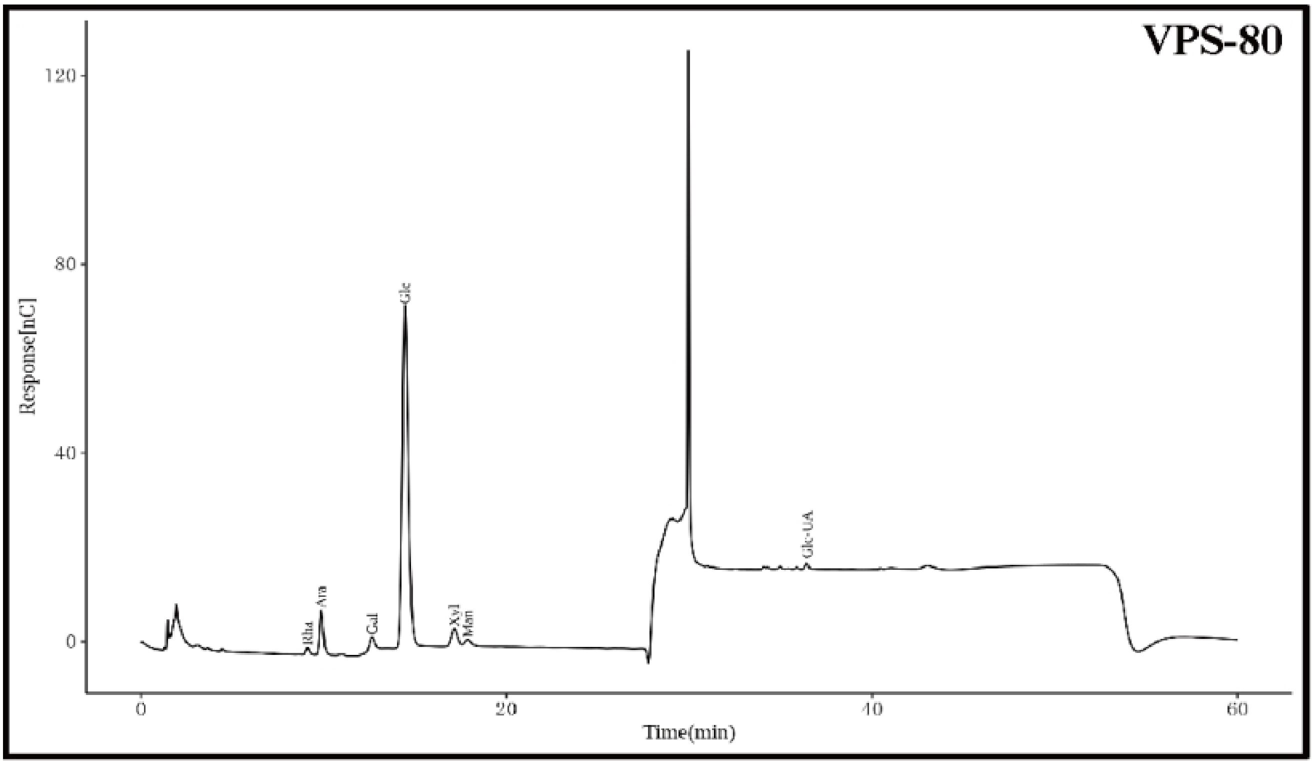

Figure S1 The monosaccharide composition of VPS components.

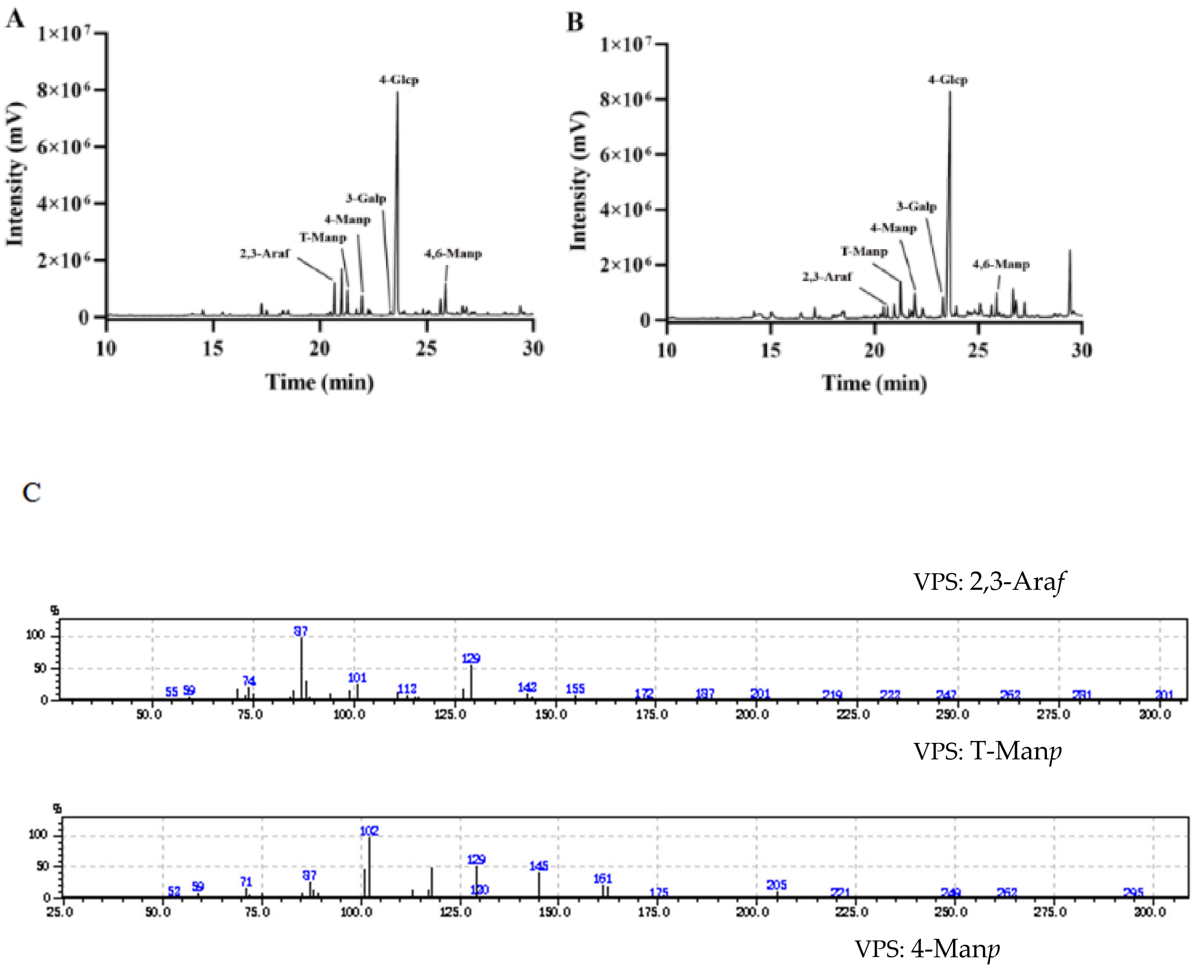

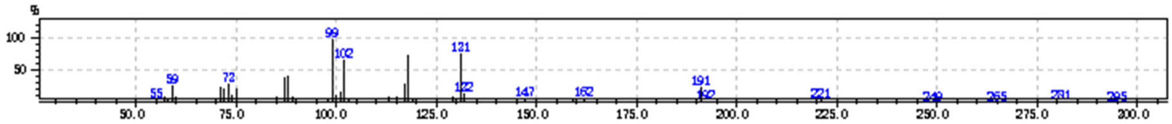

VPS:3-Gal*p*

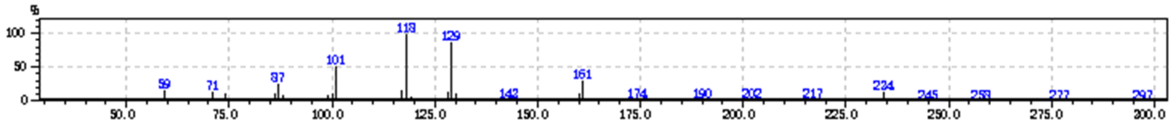

VPS: 4-Glc*p*

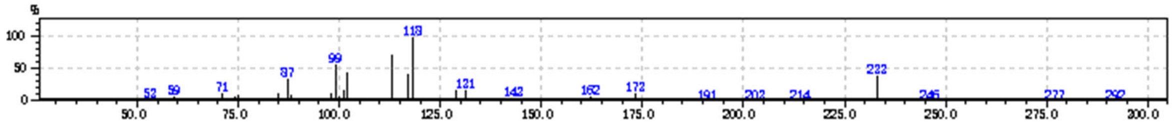

VPS: 4,6-Man*p*

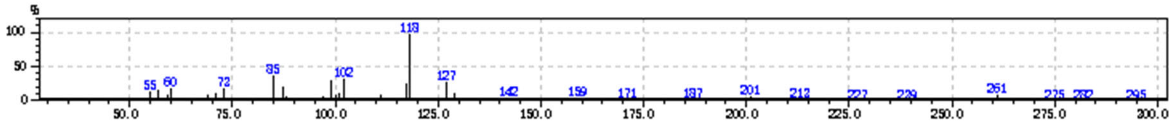

VPS-30: 2,3-Ara*f*

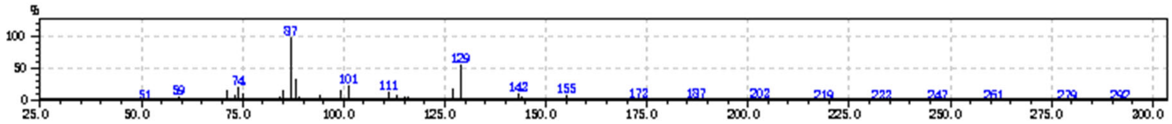

VPS-30: T-Man*p*

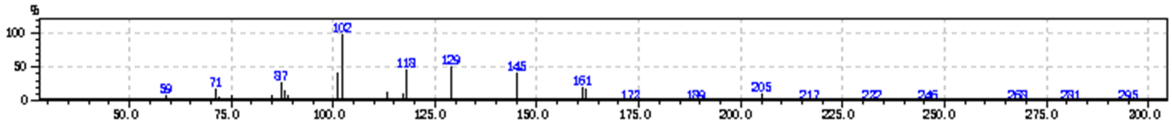

VPS-30: 4-Man*p*

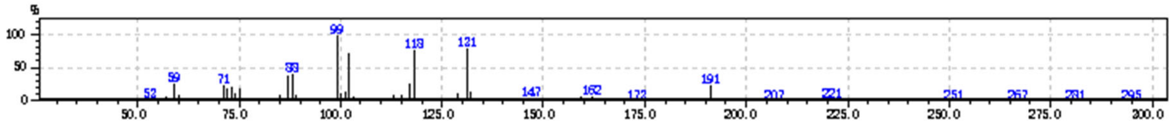

VPS-30: 3-Gal*p*

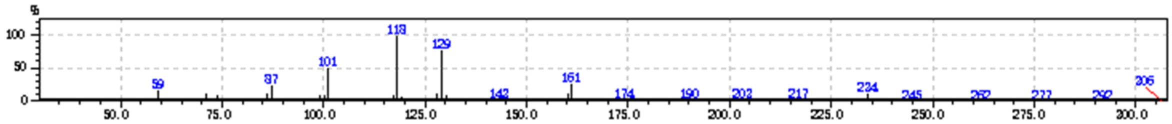

VPS-30: 4-Glc*p*

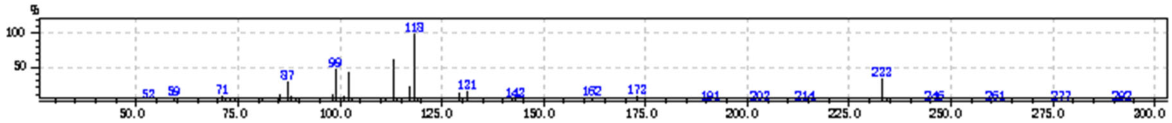

VPS-30: 4,6-Man*p*

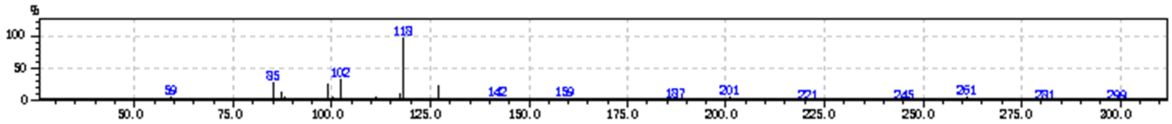

Figure S2 Total Ion chromatogram of PMAA of VPS and VPS-30, and the mass spectra of each PMAA. (A) Total Ion chromatogram of PMAA of VPS. (B) Total Ion chromatogram of PMAA of VPS-30. (C) The mass spectra of each PMAA.

A

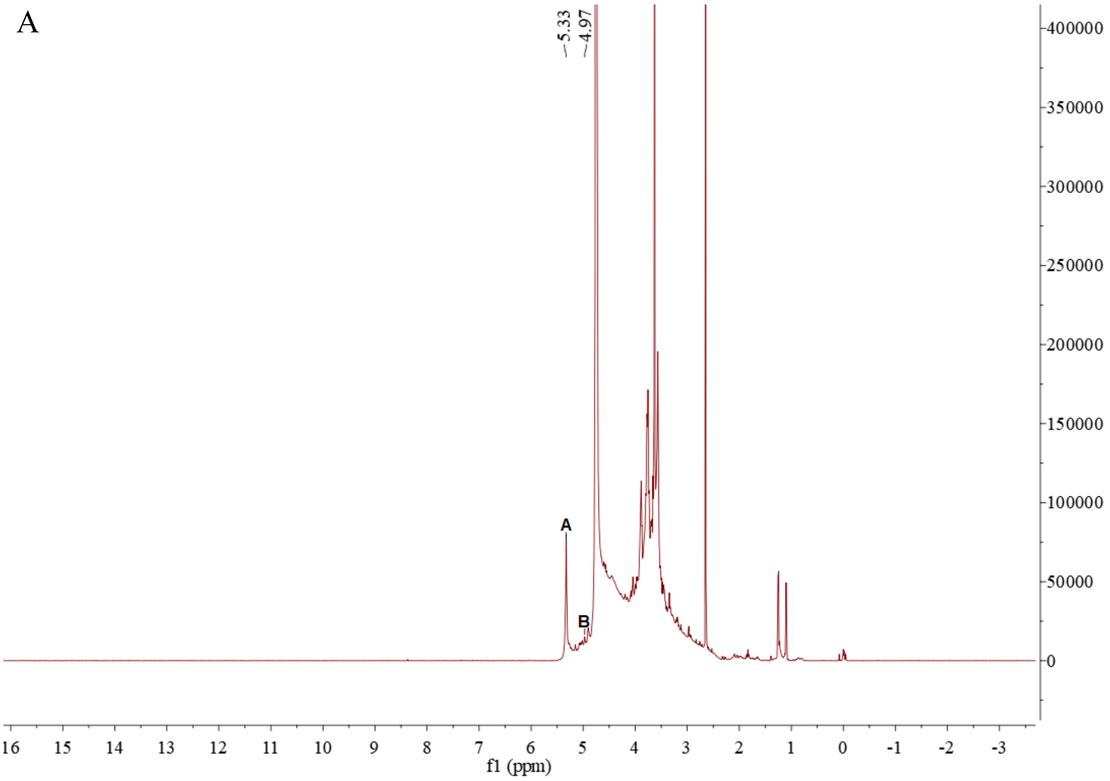

B

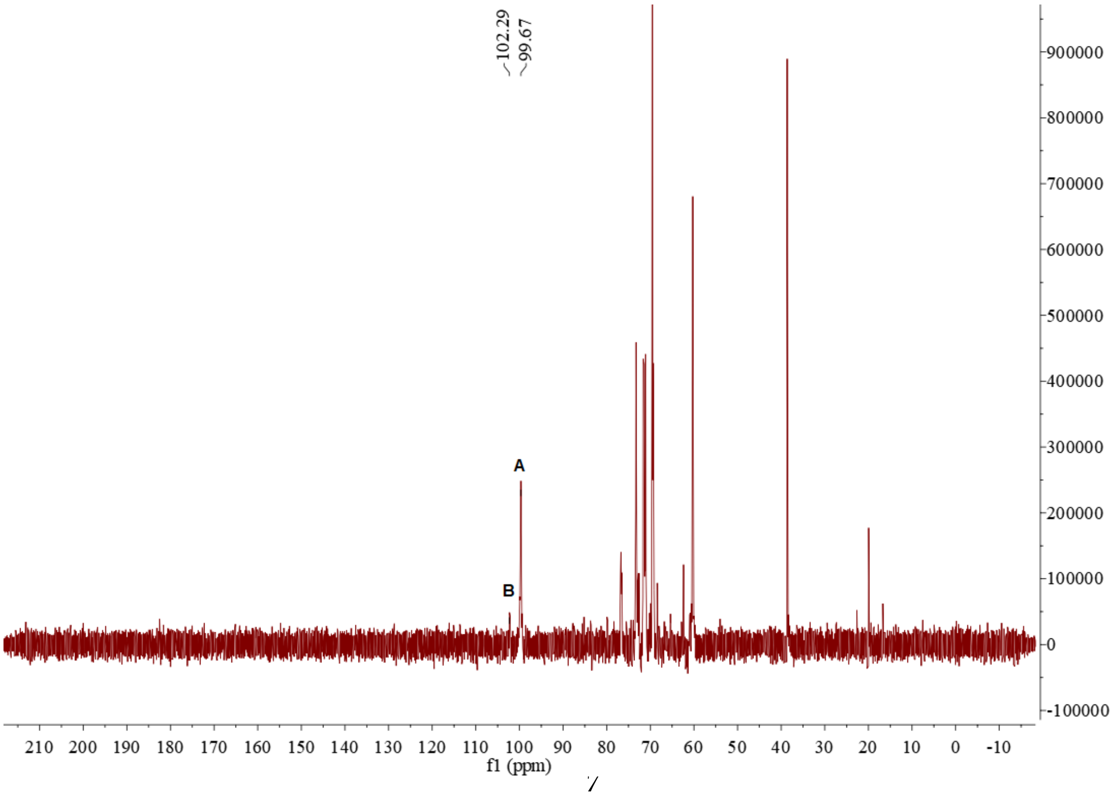

C

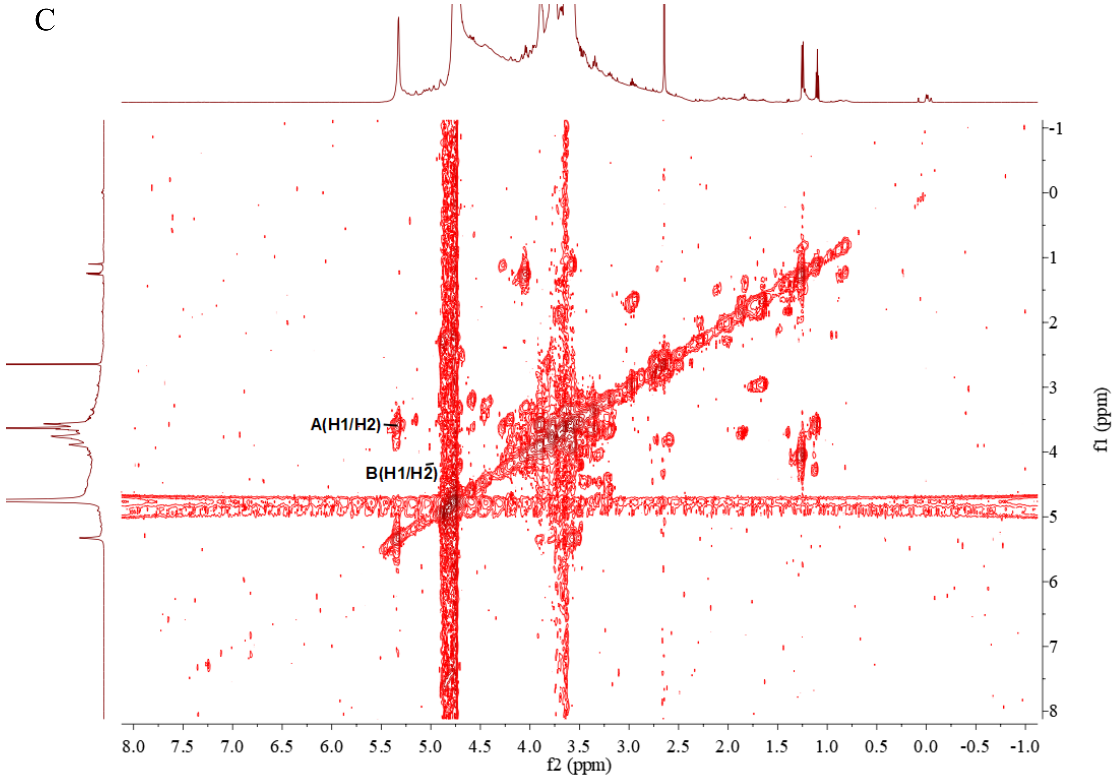
A(H1/H2)
B(H1/H2)
f1 (ppm)
f2 (ppm)

D

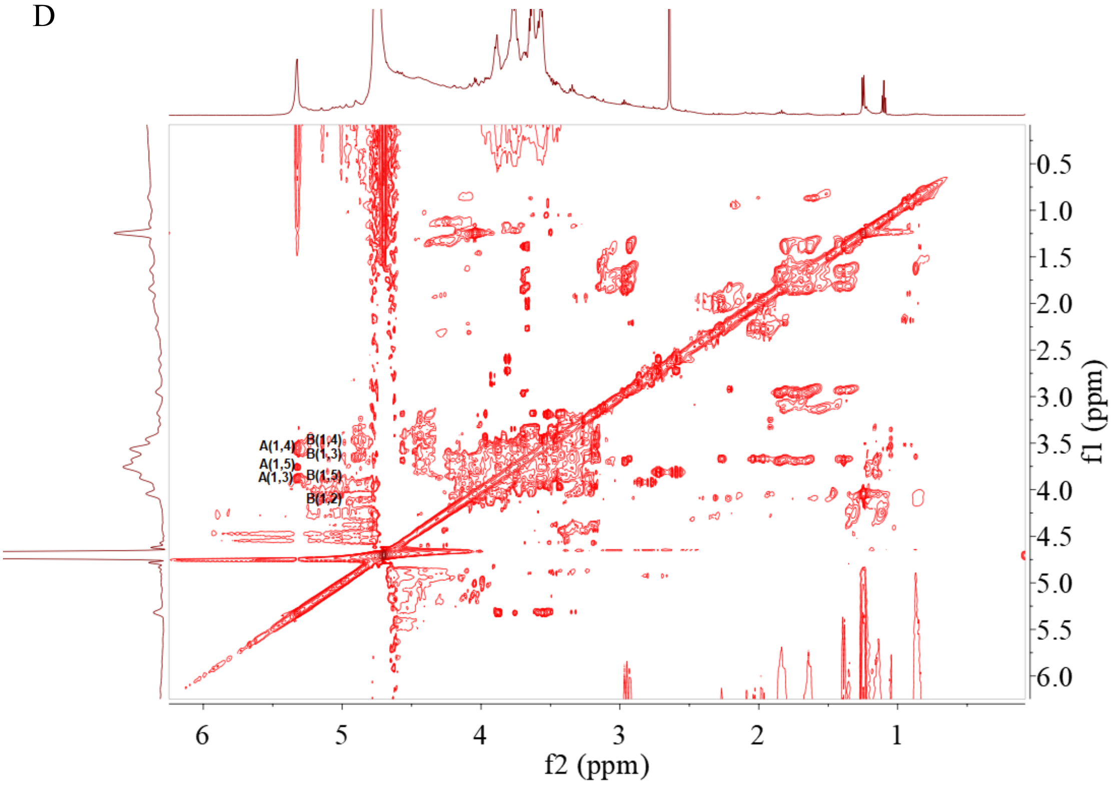
A(1,4)
B(1,4)
B(1,3)
A(1,5)
A(1,3)
B(1,5)
B(1,2)
f1 (ppm)
f2 (ppm)

E

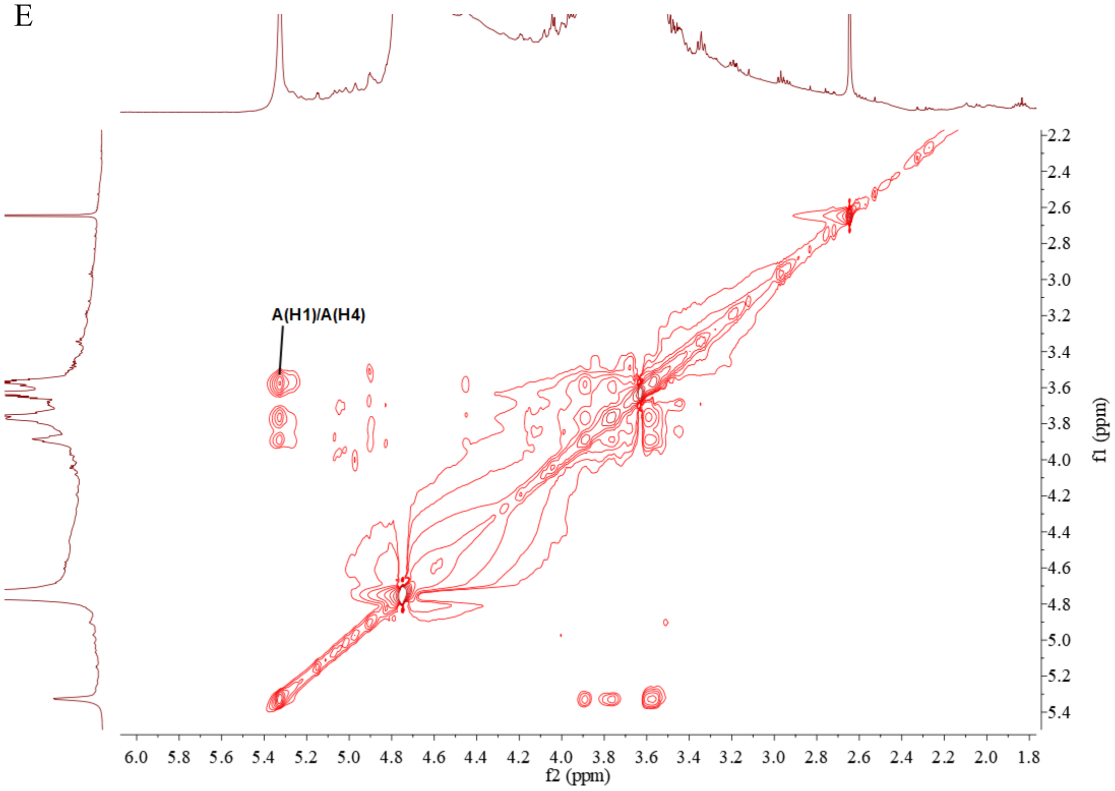

Figure S3. NMR spectra of VPS-30. (A) ^{1}H-NMR spectrum. (B) ^{13}C -NMR spectrum. (C) ^{1}H-^{1}H COSY spectrum. (D) TCOSY spectrum. (E) NOSEY spectrum.
